# Consensus Approach for Standardizing the Screening and Classification of Preterm Brain Injury Diagnosed With Cranial Ultrasound: A Canadian Perspective

**DOI:** 10.3389/fped.2021.618236

**Published:** 2021-03-08

**Authors:** Khorshid Mohammad, James N. Scott, Lara M. Leijser, Hussein Zein, Jehier Afifi, Bruno Piedboeuf, Linda S. de Vries, Gerda van Wezel-Meijler, Shoo K. Lee, Prakesh S. Shah

**Affiliations:** ^1^Department of Pediatrics, University of Calgary, Calgary, AB, Canada; ^2^Departments of Diagnostic Imaging and Clinical Neurosciences, University of Calgary, Calgary, AB, Canada; ^3^Department of Pediatrics, Dalhousie University, Halifax, NS, Canada; ^4^Department of Pediatrics, Université Laval and Centre de recherche du CHU de Québec – Université Laval, Quebec City, QC, Canada; ^5^Department of Neonatology, Wilhelmina Children's Hospital, University Medical Center Utrecht, Brain Center, University Utrecht, Utrecht, Netherlands; ^6^Isala Women and Children's hospital, Zwolle, Netherlands; ^7^Department of Pediatrics, Mount Sinai hospital, Toronto, ON, Canada; ^8^Department of Pediatrics, University of Toronto, Toronto, ON, Canada

**Keywords:** preterm (birth), brain injury, cranial ultrasonography, intraventriclar hemorrhage, post-hemorrhagic ventricular dilatation

## Abstract

Acquired brain injury remains common in very preterm infants and is associated with significant risks for short- and long-term morbidities. Cranial ultrasound has been widely adopted as the first-line neuroimaging modality to study the neonatal brain. It can reliably detect clinically significant abnormalities that include germinal matrix and intraventricular hemorrhage, periventricular hemorrhagic infarction, post-hemorrhagic ventricular dilatation, cerebellar hemorrhage, and white matter injury. The purpose of this article is to provide a consensus approach for detecting and classifying preterm brain injury to reduce variability in diagnosis and classification between neonatologists and radiologists. Our overarching goal with this work was to achieve homogeneity between different neonatal intensive care units across a large country (Canada) with regards to classification, timing of brain injury screening and frequency of follow up imaging. We propose an algorithmic approach that can help stratify different grades of germinal matrix-intraventricular hemorrhage, white matter injury, and ventricular dilatation in very preterm infants.

## Introduction

Brain injury remains a frequent and clinically significant problem in very preterm infants born at or before 32 weeks' gestation (1). Reported incidence rates are 5.8% to 6.1% for grade 3 intraventricular hemorrhage (IVH) and periventricular hemorrhagic infarction (PVHI), and 12% for grades 1 and 2 IVH (2) using Papile's classification (3). Different types of lesions can affect the preterm infant's brain including hemorrhagic lesions, ischemic lesions or maturation arrest (4). Cranial ultrasound (cUS) is an easily accessible and widely used tool to detect these lesions. However, small intraparenchymal hemorrhages and small ischemic lesions can sometimes be difficult to detect or differentiate using cUS. In addition, some forms of brain injury including mild ischemic changes, can be difficult to distinguish from changes caused by normal maturational phenomena. Magnetic resonance imaging (MRI) offers greater sensitivity and specificity and enhanced lesion characterization. However, at present MRI remains impractical as a routine screening tool, and at times this technique identifies subtle lesions with unproven significance. Sequential cUS during the neonatal period can reliably identify many brain abnormalities enabling the prediction of abnormal neurodevelopmental outcomes at 1 year and 3 years of postnatal age (5–7). Thus, cUS remains the universal screening tool to identify and monitor brain injury in preterm neonates.

The most common brain injury types in the preterm neonate detected by cUS include blood in the germinal matrix and/or lateral ventricles (germinal matrix hemorrhage-intraventricular hemorrhage, GMH-IVH); hemorrhagic or ischemic lesions in the cerebral white matter (parenchymal lesions); post-hemorrhagic ventricular dilatation (PHVD); ventricular dilatation without hemorrhage (ex-vacuo); posterior fossa hemorrhage (cerebellar or extracerebellar hemorrhage); focal infarcts; subdural hematoma; and cerebral venous sinus thrombosis. In most neonatal intensive care units (NICUs), it is accepted practice to perform serial cUS examinations throughout the neonatal period in infants born at less than 32 weeks' gestational age (GA). The purpose of such screening is to identify brain injury, as this has been associated with adverse neurodevelopmental outcomes in very preterm infants (8–10). Unfortunately, significant variability in classification and definition of the different types of preterm brain injury can lead to challenges or inaccuracies when studying interventions or risk factors using these outcome measures (11, 12).

The Canadian Neonatal Network (CNN) and Canadian Preterm Birth Network (CPTBN) are groups of researchers across Canada with the common goal of conducting and leading multi-disciplinary, collaborative research dedicated to improving neonatal-perinatal health and healthcare in Canada and internationally. The CNN maintains a standardized national neonatal database that provides a unique opportunity for researchers to participate in collaborative projects on national and international scales. Along with benchmarking activities, the Network members are actively involved in national quality improvement initiatives through the Evidence-based Practice for Improving Quality (EPIQ) program – one goal of which is to decrease the incidence of acute brain injury using quality improvement methodology (13). In order to understand the pathological processes underlying preterm brain injury such that modifiable factors can be addressed, it was important to first undertake an elaborate process of homogenizing the classifications and descriptions for preterm brain injury across reporting centers within the country. We planned this standardization initiative with the aim to disseminate our approach so it can be used in the future for national and international benchmarking, clinical care quality improvement, and risk factor identification purposes.

## Materials and Methods

The preterm infant's brain is both susceptible and vulnerable to hemorrhagic and ischemic injury and their various secondary complications. A more detailed description of the pathogenesis that leads to neonatal brain injury is beyond the scope of this article, and is extensively addressed in excellent work from other researchers (14, 15). In this project, our objective was to use the combined expertise of neonatal and radiology experts across Canada to develop a standardized approach for (a) classification of the different forms of IVH; (b) measurement of ventricular size; (c) diagnosis of white matter injury (WMI) including both echodensities and echolucencies (cystic periventricular leukomalacia or porencephaly); (d) diagnosis of cerebellar hemorrhage (CBH); and (e) definition of brain injury determined by cUS. Our overarching goals were to facilitate consistent practice patterns for defining brain injury; and to reduce inter-individual, intra-center, and inter-center variation in cUS screening, diagnosis, and grading of brain injury in preterm infants. The scope of this project was limited to producing a consensus document outlining limitations of the current approach and providing guidelines for conducting cUS examinations and defining preterm brain injury.

We created a task force of interested experts representing both radiology and neonatology departments from all centers participating in the CNN (31 tertiary care NICUs and their affiliated radiology departments). The task force met regularly, performed critical literature review, and created and conducted online surveys to identify and confirm sources of variation in the definitions of preterm brain injury. This groundwork was used to plan and conduct two hands-on workshops using locally developed (Calgary, Canada) and validated cUS simulators specifically for the project (16). The simulators were loaded with a library of cases representing sources of variability. The experts in neonatology and radiology were given opportunities to discuss the cases and debate their different viewpoints at hands-on small group stations. At the end of each workshop, a variety of cases were discussed by the whole group using an interactive voting system. Following the workshops, task force members wrote the first draft of this article and then carried out several rounds of electronic discussion on that initial draft and this final version. The suggested approach to classification and timing of brain scans presented in this paper was also reviewed by national and international experts in the fields of neonatal neurology and neuroimaging. We used the hierarchy of evidence from the Centre for Evidence-Based Medicine (Oxford CEBM-March 2009) to rate our recommendations (17).

Two questions were answered through this consultative process:

- What are the standardized definitions of common types of brain injury (a-d) in preterm infants as visualized with cUS and how can we differentiate these injury types among them and from other types of preterm brain injury?
- What is the recommended timing for evaluation and screening of brain injury in preterm neonates using cUS?

## Results

Standardizing definitions of common types of preterm brain injury:

### Acute or Early Injury to Preterm Brain

Acute injury to the preterm brain occurs in the form of hemorrhage or infarction and may or may not be associated with ventricular dilatation.

#### Hemorrhage

GMH-IVH in preterm infants originates from the germinal matrix and can either be restricted to the germinal matrix or extend into the lateral ventricles. The original grading classification described by Papile (3), and later adapted by Volpe (18) with minor modifications, is still commonly used in clinical practice. The classification is based on the amount and extension of hemorrhage into the lateral ventricles and the presence of acute ventricular dilatation, and is a strong predictor of neurodevelopmental outcomes (8). GMH-IVH as detected by cUS and possible associated acute dilatation related to the mass effect of a large hemorrhage occurs mostly in the first 3 days after birth (i.e., within the “critical window”). The cUS image with the greatest severity of GMH-IVH in the first week after birth (in case more than one study was performed) should be considered for detection and initial grading purposes. Ventricular dilatation with IVH, referred to as PHVD, mostly occurring 7 to 10 days (up to 2-3 weeks) after the occurrence of GMH-IVH and identified after the first week from birth, should be considered a complication of the early hemorrhagic injury (Level of Evidence 1b).

#### Germinal Matrix-Intraventricular Hemorrhage Gradings

The early grading system proposed by Papile (3), and later adapted by Volpe (18), classified GMH-IVH into three grades and included a separate notation for the presence of periventricular hemorrhagic infarction (PVHI). These grading systems are well acknowledged; thus, for consistency with the established literature, no modifications were made in our consensus viewpoint except for the addition of anterior horn width (AHW) measurement.

The three principle imaging elements used to grade GMH-IVH are (1) detection of hemorrhage (confined echogenicity on cUS) in the germinal matrix or within the ventricular system, (2) measurement of ventricular enlargement that defines ventricular dilatation, and (3) assessment of periventricular white matter for abnormal echodensities that may represent either ischemic or hemorrhagic parenchymal injury. These principle imaging elements are used in the four main steps that will aid in the classification of GMH-IVH (Table 1).

**Table 1:** Steps for classifying GMH-IVH in preterm infants (Figures 3–9).

Steps for Classifying GMH-IVH in Preterm Infants
STEP #1: Is there hemorrhage within/around the germinal matrix AND/OR within the lateral, third, or fourth ventricle?
NO: Report negative for GMH-IVH, go to Step #4.
YES: Report positive for GMH-IVH, go to Step #2.
STEP #2: Is the hemorrhage confined to the germinal matrix region?
NO: Blood is detected in the lateral ventricle or on the choroid plexus = Grade II or III GMH-IVH, go to Step #3.
YES: Either common (Image 3) or uncommon (Image 4) in location AND no blood in lateral ventricle = Grade I GMH-IVH, go to Step #4.
STEP #3: Is the IVH distending the ipsilateral lateral ventricle AND the AHW measures >6 mm?
NO: Grade II GMH-IVH (Images 5,6), go to Step #4.
YES: Grade III GMH-IVH (Image 7), go to Step #4.
STEP #4: Is focal echogenicity present in the periventricular white matter adjacent (i.e., ipsilateral) to the side of GMH-IVH or, in case of bilateral GMH-IVH, ipsilateral to the largest GMH-IVH?
NO: If negative for GMH-IVH in Step #1, report as normal.
NO: If positive for GMH-IVH in Step #1, report as defined in Steps #1-3.
YES: Is there Grade I, II, or III GMH-IVH present?
If no GMH-IVH present, report as ischemic injury (Image 8). If GMH-IVH present, report as PVHI (Image 9 and Table 1).

*GMH-IVH: germinal matrix hemorrhage-intraventricular hemorrhage. PVHI: periventricular hemorrhagic infarction; AHW: anterior horn width*.

The steps outlined in Table 1 have been integrated into a smartphone application and made freely available for iPhones and android devices (https://apps.apple.com/ca/app/hus-diagnostic/id1527886689) and on google play https://play.google.com/store/apps/details?id=com.husdiagnostic&hl=en&gl=US) to increase user compliance with the new classification system.

**Figure 1:**
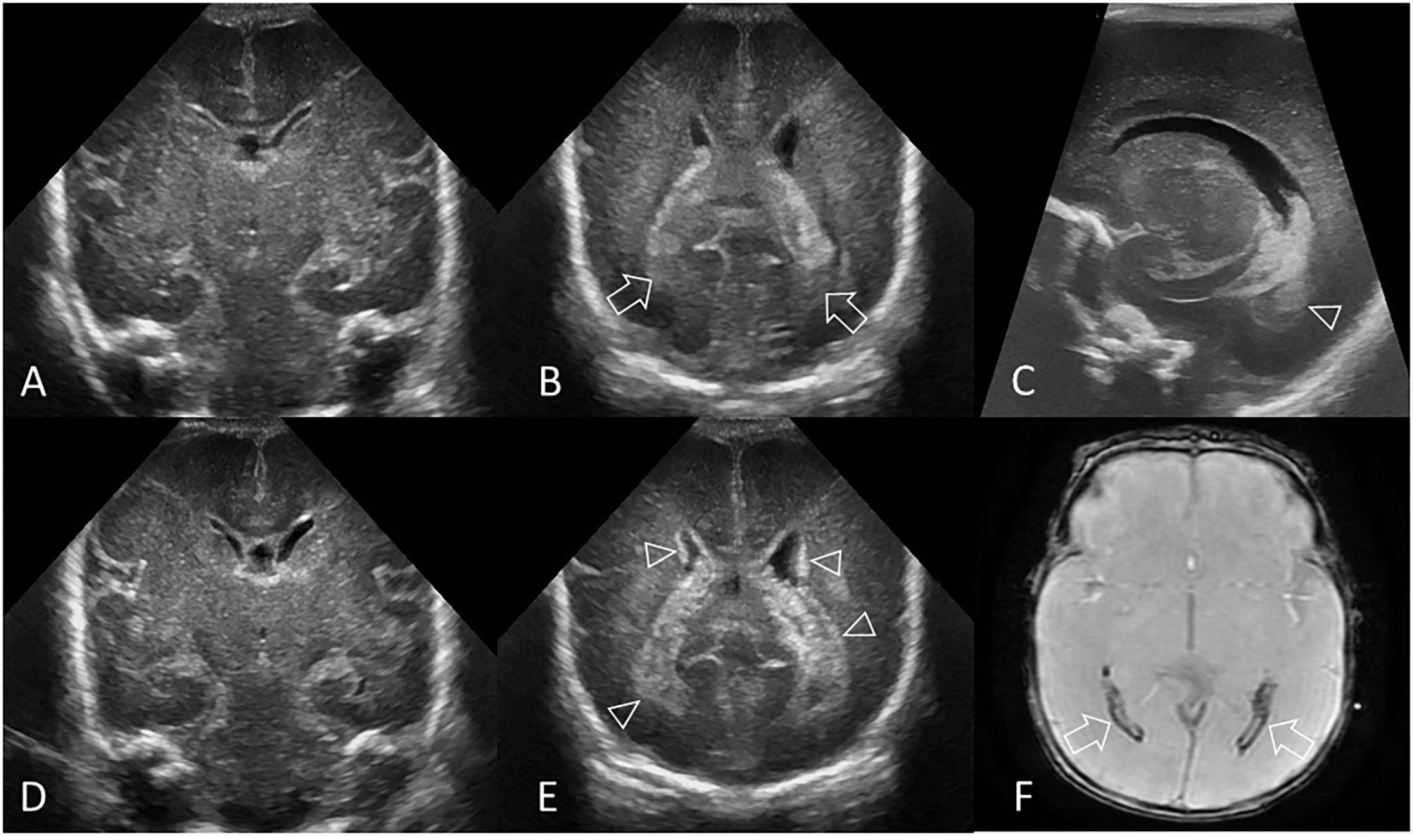
Hyperechogenic ependyma as indirect sign of IVH. Ultrasound and MRI scan in a preterm infant (GA 24 weeks 3 days). **(A)** Coronal scan at the level of the frontal horns of the lateral ventricles shows no evidence of GMH, IVH, or ventricular enlargement. **(B)** Coronal scan at the level of the trigone of the lateral ventricles shows a small volume of echogenic clot within the posterior body of both ventricles (arrows); this finding was not recognized at time of original reporting. **(C)** Parasagittal scan through the left lateral ventricle also shows the small volume IVH (arrowhead). **(D)** Follow up coronal scan 14 days after birth at the level of the frontal horns still does not show clear evidence of germinal matrix clot. **(E)** Coronal scan at the level of the trigone of the lateral ventricles shows new hyperechogenic ependyma involving both lateral ventricles (arrowheads), with more extensive IVH within the bodies of the lateral ventricles. No hydrocephalus has developed. The presence of this hyperechoic ependyma on the follow-up scan facilitated a retrospective diagnosis of grade II GMH-IVH in this infant. **(F)** Axial T2^*^ gradient-echo MR image 3 months after birth shows increased susceptibility effect (hypointense signal) that outlines the occipital horns of both lateral ventricles (arrows) from hemosiderin/ferritin and confirms presence of earlier IVH. Several microhemorrhages were also identified in the bilateral germinal matrix regions on this blood sensitive MR sequence (not shown).

Echodensity on cUS is defined as areas of “brightness” of higher intensity than the choroid plexus. Recent hemorrhage is echogenic, although ultrasonographic characteristics change over time; the clot becomes less echogenic and will liquefy and become echolucent. It is important to evaluate the occipital horns of the lateral ventricles for any dependent blood layering. Cerebrospinal fluid in the occipital horn posterior to the calcarine fissure should not contain any choroid plexus. Echogenicity that mimics blood can, however, occasionally be seen in the occipital horn on parasagittal views using the anterior fontanel, but should be interpreted as artifactual if not seen in two different imaging planes. Posterior fontanel approach cUS may help to distinguish such artifact from hemorrhage in the occipital horn if there is uncertainty. Blood (echogenicity) within the fourth ventricle is most easily identified using the mastoid window. The identification of echogenic ependyma (Figure 1) can be a helpful indirect sign to indicate that intraventricular blood of subacute duration is present (19, 20). The presence of an echogenic ependymal wall can be seen 10-14 days after an IVH and will allow for a retrospective diagnosis of IVH and may indicate an antenatal timing of IVH if identified in the first few days after birth (19, 20). Of note, echogenic ependyma can also be a marker of meningitis.

The traditional approach to grade GMH-IVH is to consider bilateral GMH-IVH as unilateral injury, and only record the side with the worst grade in most study's analysis. This more simplified approach may be flawed since bilateral GMH-IVH is equal or more prevalent than unilateral GMH-IVH, and the likelihood of a poor outcome increases with bilateral injury. Improved prognostication of neurodevelopmental outcome post GMH-IVH has been reported in preterm infants based upon laterality of brain injury (21). The independent grading of GMH-IVH for the right and left cerebral hemisphere is thus considered to be more informative and is recommended for use in our consensus viewpoint.

##### Grade I GMH-IVH

**Figure 2:**
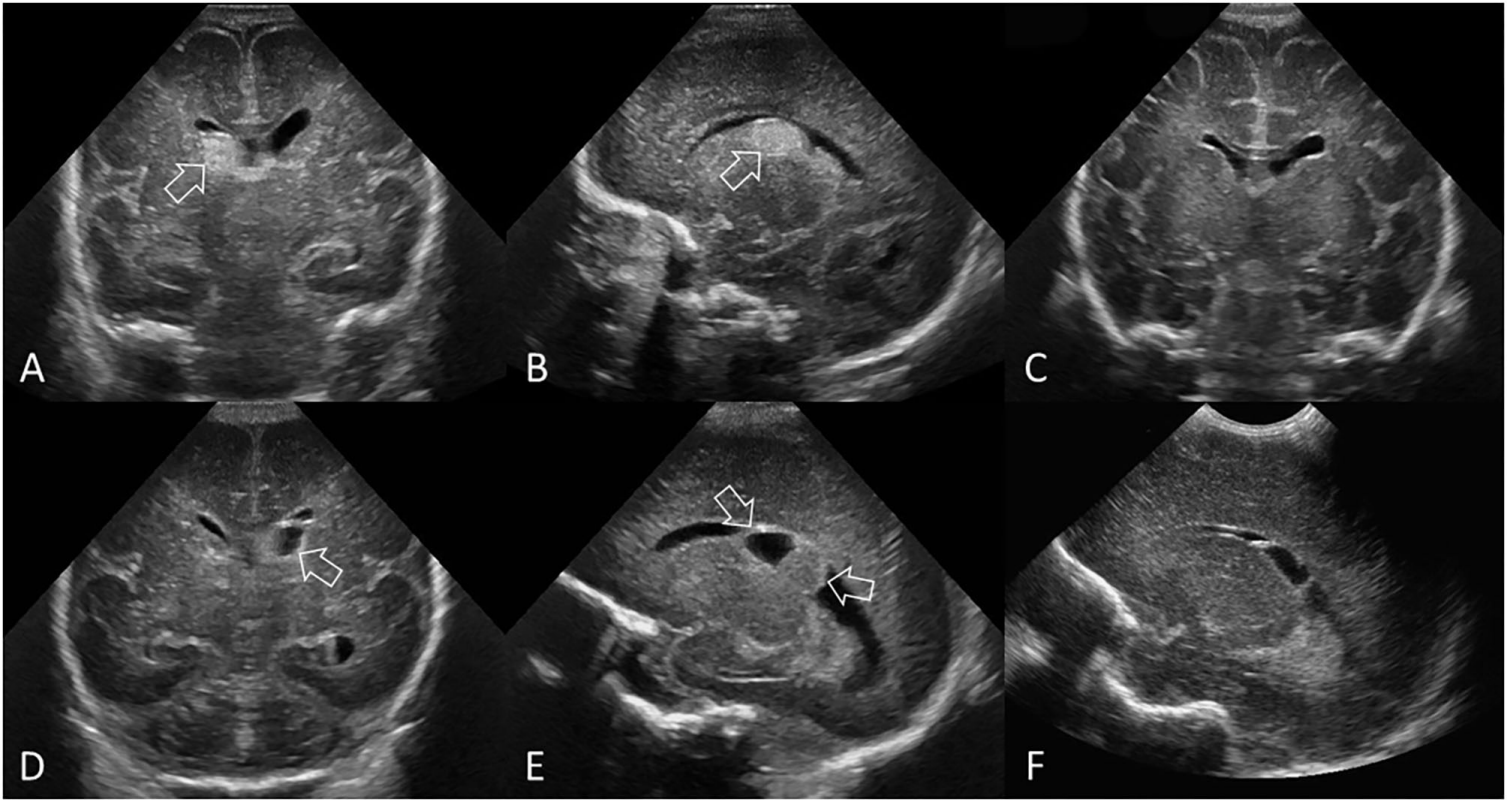
Grade I GMH-IVH. **(A–C)** Ultrasound scan in a preterm infant (GA 26 weeks) with common pattern of grade I GMH-IVH. **(A)** Coronal scan at the level of the frontal horns of the lateral ventricles. **(B)** Parasagittal scan through the right lateral ventricle. The location of the echogenic clot (arrow) at the right caudothalamic notch is typical of grade I GMH-IVH. There is no IVH or ventricular dilatation. **(C)** Follow up coronal scan 12 weeks later shows complete resolution of the GMH. **(D–F)** Ultrasound scan in a different preterm infant (GA 27 weeks) with a less common location of grade I GMH-IVH. **(D)** Coronal scan at the level of the frontal horns of the lateral ventricles. **(E)** Parasagittal scan through the left lateral ventricle. The clot (arrows) is centered posterior to the left caudothalamic notch, but is still considered a grade I GMH-IVH as there appears to be no blood within the ventricles. The clot is non-uniform in its echogenicity, with the echolucent portion representing cystic degeneration (liquefaction) typical of an “older” GMH-IVH at this stage. There is no ventricular dilatation. **(F)** Follow-up parasagittal scan 9 weeks later shows further cystic degeneration and smaller clot size.

Typically, hemorrhage confined to the germinal matrix is classified as grade I GMH-IVH or GMH (Figures 2A–C). There is no blood in the ventricles or within the choroid plexus. It can be small or large. Larger-sized grade I GMH-IVH may compress the foramen of Monro (because of its relatively close proximity to the caudothalamic notch) and cause (partial) ipsilateral ventricular outflow obstruction. A less common location of a grade I GMH-IVH is centering posterior to the caudothalamic notch (Figures 2D–F). Evolution of a grade I GMH-IVH includes clot retraction and a progressively hypoechoic appearance on serial cUS. Larger hemorrhages may eventually liquefy, leaving cystic degeneration changes within the clot (Figures 2D–F). It can be difficult to confidently differentiate some acquired (post-hemorrhagic) subependymal cysts from congenital (germinolytic) cysts as both may be hemorrhagic.

##### Grade II IVH

**Figure 3:**
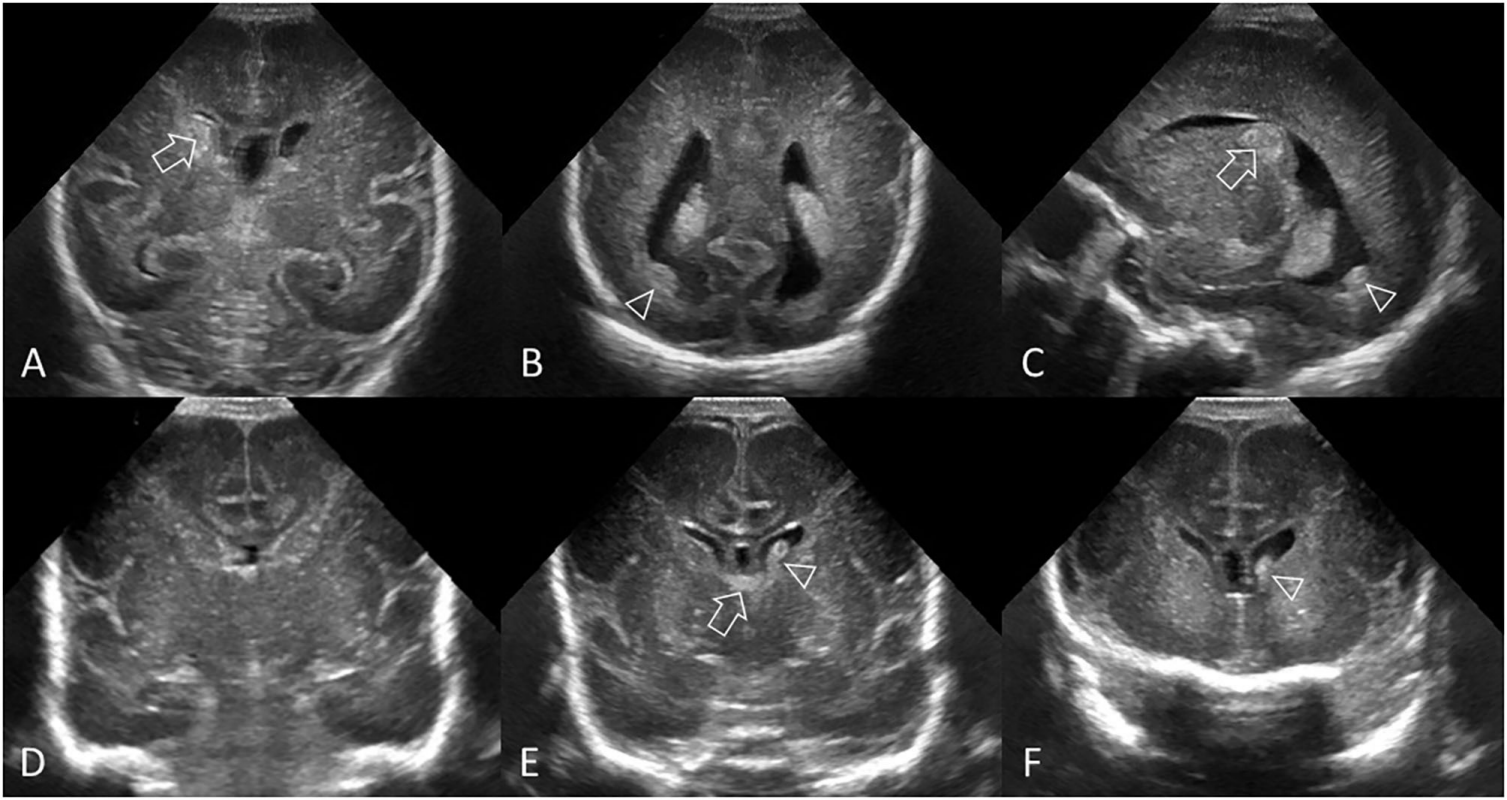
Grade II GMH-IVH. **(A–C)** Ultrasound scan in a preterm infant (GA 26 weeks) with common pattern of grade II GMH-IVH. **(A)** Coronal scan at the level of the frontal horns of the lateral ventricles shows echogenic clot involving the caudate nucleus (arrow). No ventricular dilatation. **(B)** Coronal scan at the level of the trigone of the lateral ventricles shows a small volume of intraventricular clot layering dependently (arrowhead). **(C)** Parasagittal scan through the right lateral ventricle shows the same clot centered at the caudothalamic notch (arrow) and the intraventricular hemorrhage within the occipital horn separate to choroid plexus (arrowhead). **(D–F)** Ultrasound scan in a different preterm infant (GA 27 weeks) with delayed pattern of Grade II GMH-IVH. **(D)** Coronal scan, at the level of the frontal horns of the lateral ventricles, was initially normal 2 days after birth. No GMH-IVH was detected. **(E)** Follow-up coronal scan 6 days after birth shows echogenic clot in the left lateral ventricle (arrowhead). The AHW is <6 mm (measurement not shown). Normal choroid plexus in the roof of the third ventricle (arrow). **(F)** Additional coronal view of the frontal horns anterior to the foramen of Monro shows further echogenic intraventricular clot (arrowhead). There is no extension of choroid plexus anteriorly past the caudothalamic grooves (i.e., the frontal and occipital horns have no choroid plexus), thus echogenic material in these sites suggests IVH. This infant had two cUS exams in the first week after birth. The cUS scan showing the greatest severity of GMH-IVH in the first week (i.e., cUS at 6 days after birth in this case) should be considered for GMH-IVH classification purposes.

The presence of hemorrhage (echogenicity) in the lateral ventricle without acute ventricular dilatation is classified as grade II GMH-IVH (Figure 3). Most cUS examples will show clear spill-over of blood (echogenic area) from a GMH into the ventricle. The intraventricular clot in grade II GMH-IVH characteristically fills less than 50% of the lateral ventricle.

##### Grade III IVH

**Figure 4:**
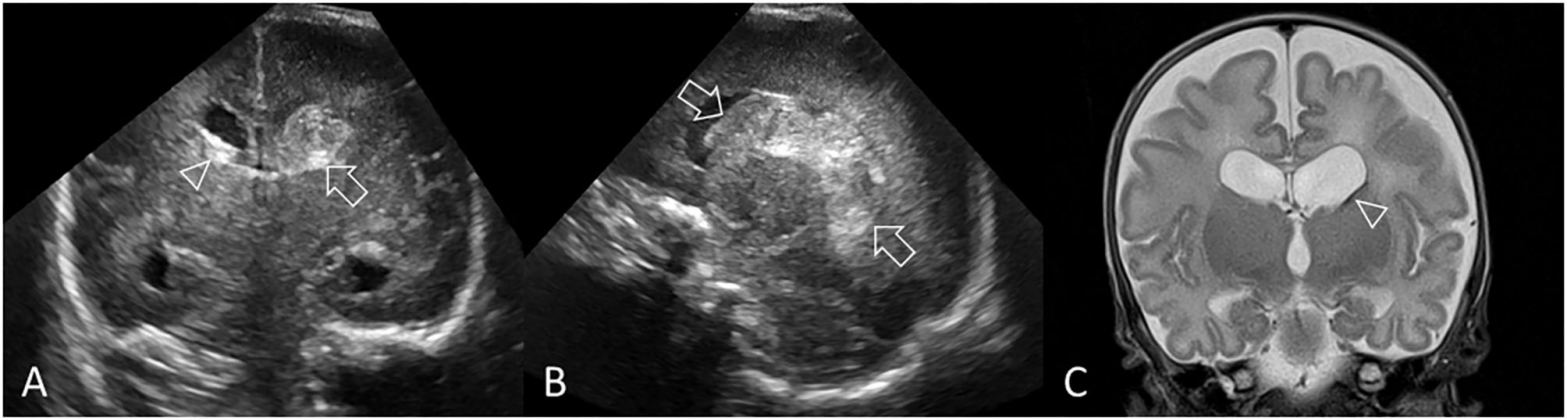
Left-sided grade III GMH-IVH. Ultrasound scan in a preterm infant (GA 25 weeks) showing bilateral IVH. **(A)** Coronal scan, at the level of the bodies of the lateral ventricles, 2 days after birth shows a large left GMH-IVH (arrow) with intraventricular blood acutely distending its lateral ventricle (AHW = 7 mm, measurement not shown). Note also the enlarged temporal ventricular horns. A small amount of blood in the right lateral ventricle (arrowhead) is present. As there is no clear GMH on the right, it is likely that the blood in the right lateral ventricle has flowed, or re-distributed, from the left-sided GMH-IVH. There is ballooning (rounding) of the right frontal ventricular horn (AHW = 6.5 mm, measurement not shown). **(B)** Parasagittal view shows that the left grade III GMH-IVH fills >50% of the distended lateral ventricle. **(C)** Coronal T2-weighted MR image 3 months after birth shows subtle hypointense staining at the left caudothalamic notch from hemosiderin/ferritin (arrowhead). No hypointense signal involves the right germinal matrix region, consistent with the earlier ipsilateral IVH having represented “spill-over.” Widened subarachnoid spaces and persistent ventriculomegaly mostly reflects tissue volume loss.

Hemorrhage (echogenicity) that spills over into the ventricle and causes acute ventricular dilatation (AHW >6 mm) is classified as grade III GMH-IVH (Figure 4). The volume of intraventricular clot in grade III GMH-IVH typically fills 50% or more of one or both lateral ventricles and increases the risk of ventricular dilatation.

##### Periventricular Hemorrhagic Infarction

**Figure 5:**
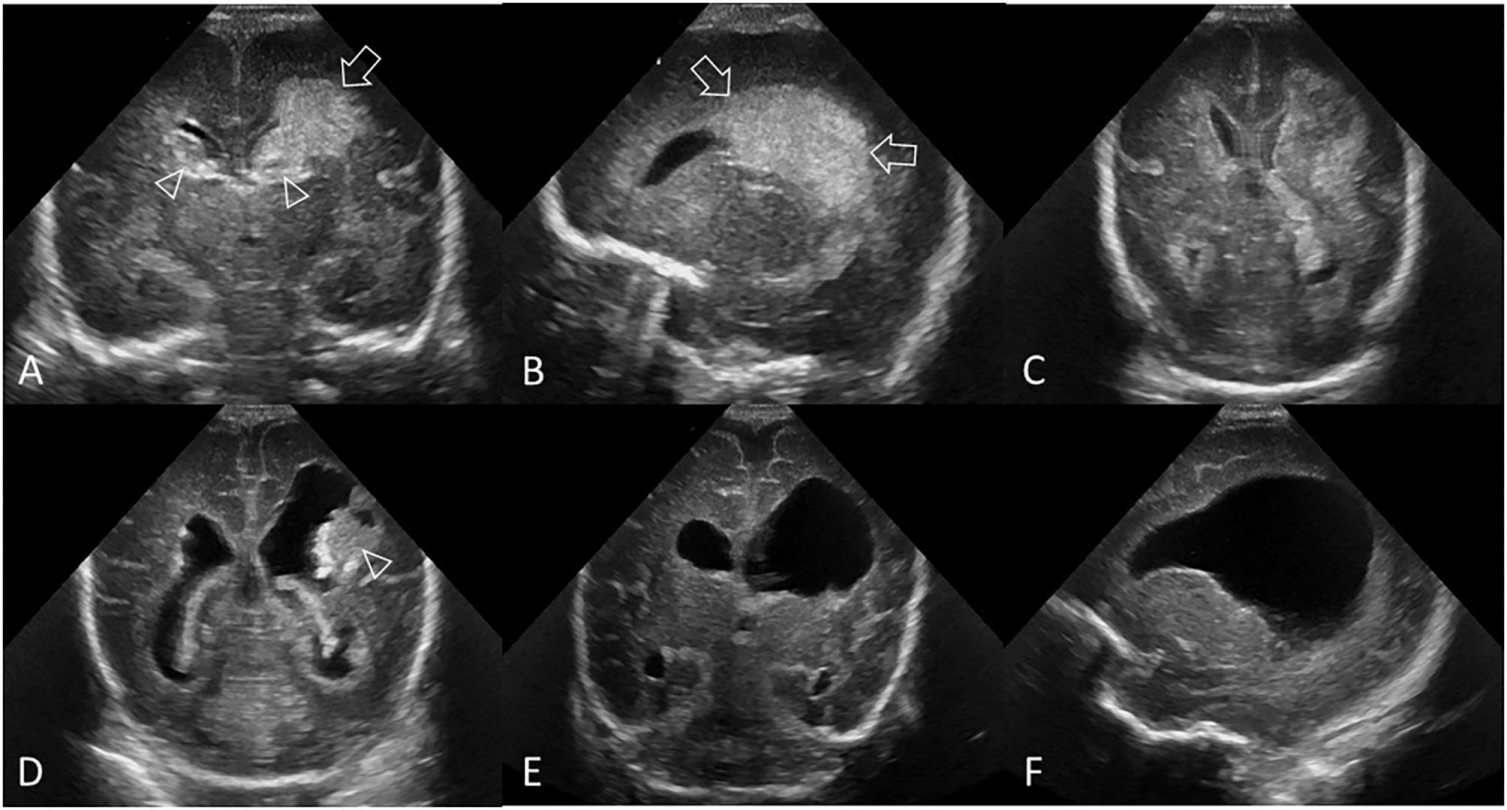
Grade III GMH-IVH + PVHI. Ultrasound scan in a preterm infant (GA 24 weeks) with bilateral GMH-IVH and left-sided PVHI. **(A)** Coronal scan 2 days after birth showing a left-sided grade III GMH-IVH (arrowhead, AHW = 7 mm, measurement not shown) and large echodensity in the left frontoparietal white matter (arrow). Smaller right-sided grade II GMH-IVH (arrowhead, AHW = 4 mm, measurement not shown). **(B)** Left parasagittal scan showing the periventricular echodensity (arrows) extending from the posterior frontal white matter to the parietal white matter. **(C)** Follow-up coronal scan 2 weeks after birth shows the area of PVHI beginning to develop central echolucency, indicating early cystic degeneration. **(D)** Follow-up coronal scan 7 weeks after birth shows further cystic degeneration following the PVHI with echogenic clot debris, dilated lateral ventricles, and echogenic ependymal lining. **(E,F)** Follow-up coronal and left parasagittal scans at 12 weeks show the large porencephalic cyst communicating with the lateral ventricle. Internal clot debris has cleared and ependyma is no longer echogenic. The persisting ventriculomegaly is now partially passive (i.e., ex-vacuo) in origin. In this example, the independent recording of the bilateral GMH-IVH patterns on the baseline cUS exam is recommended.

Periventricular hemorrhagic infarction (PVHI) is considered part of the injury spectrum of GMH-IVH. It most commonly occurs in very preterm infants but can still be identified in near-term and term infants. PVHI was once thought to be caused by extension of an IVH into the brain parenchyma. However, its mechanism is now considered to represent impaired drainage of medullary and terminal veins by pressure from an intraventricular clot with secondary periventricular venous congestion, white matter ischemia, and subsequent, venous hemorrhagic infarction in the periventricular white matter. PVHI occurs in approximately 15% to 20% of very preterm infants with IVH and generally develops within a few days of the onset of the GMH-IVH (9, 18). Evolution of PVHI into cavitation within the periventricular white matter shows as the hemorrhagic infarction becoming progressively echolucent on serial cUS (Figure 5). The cavitation can either result in a single porencephalic cyst or multiple cysts that completely or only partially communicate with the lateral ventricle. Some echogenic clot debris may remain within the cystic cavity for prolonged periods.

##### Cerebellar Hemorrhage

CBH is a common injury type in preterm infants (22). Recent studies have raised awareness regarding the incidence and long-term implications of CBH, which is of particular importance in extremely preterm infants (22–24). In many cases, CBH is associated with a supratentorial hemorrhage (28–71%) and blood in the fourth ventricle (25, 26). CBH is most often confined to one cerebellar hemisphere (71%) and, to a lesser extent, the vermis (20%); in the minority of cases it involves both the cerebellar hemisphere and vermis (9%) (27).

Based on the size and extent, CBHs can be divided into three groups: punctate CBH (≤ 4 mm) is usually detected on MRI and not readily seen by cUS, limited CBH (>4 mm but <1/3 of the cerebellar hemisphere), and large CBH (≥1/3 of the cerebellar hemisphere) (23). Particularly large CBHs are associated with subsequent smaller cerebellar volumes (if bilateral CBHs); or discrepant sizes of the cerebellar hemispheres, with the hemisphere being smaller at the site of CBH (if unilateral). Large CBH is associated with high mortality and morbidity, including adverse long-term neurodevelopmental and behavioral outcomes (23).

**Figure 6:**
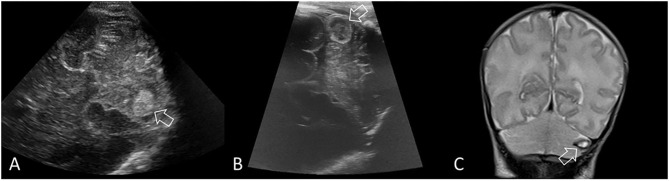
Limited cerebellar hemorrhage. Ultrasound scan in a preterm infant (GA 29 weeks) with a limited CBH (>4 mm but <1/3 of the cerebellar hemisphere). **(A)** Coronal scan through the right mastoid fontanel 5 days after birth shows a rounded echogenic lesion in the left cerebellar hemisphere (arrow), indicating intraparenchymal hemorrhage. The fourth ventricle is borderline dilated (not shown). **(B)** Repeat coronal scan through the left mastoid fontanel at 2 weeks after birth shows expected evolution of the hemorrhage (arrow), which is becoming echolucent, indicating early cystic degeneration. **(C)** Coronal T2-weighted MRI at 6 weeks after birth shows continued evolution of the hemorrhage with smaller cystic cavity that displays uniform central T2 hyperintense signal and thin hypointense rim (arrow, hemosiderin and ferritin). Surrounding cerebellar edema has resolved with local destruction and atrophy of that hemisphere. The appearance of high intensity on T1-weighted (not shown) and T2-weighted inside the hemosiderin rim stages the hemorrhage to the late subacute phase (extracellular methemoglobin).

CBH is most easily identified using the supplemental mastoid window, which allows improved visualization of the posterior fossa and midbrain structures in two planes (Figure 6). The mastoid window is also helpful for identifying hemorrhage (echogenicity) within the third or fourth ventricles (28). In cases of unexplained ventricular dilatation (i.e., without GMH-IVH), it is important to evaluate the cerebellum for any intraparenchymal hemorrhage and the fourth ventricle for acute hemorrhage and/or clot (29). CBH generally occurs in the same time window as GMH-IVH (i.e. during the first 3 days after birth), but may be identified at any time over the course of the neonatal period.

### White Matter Injury

**Figure 7:**
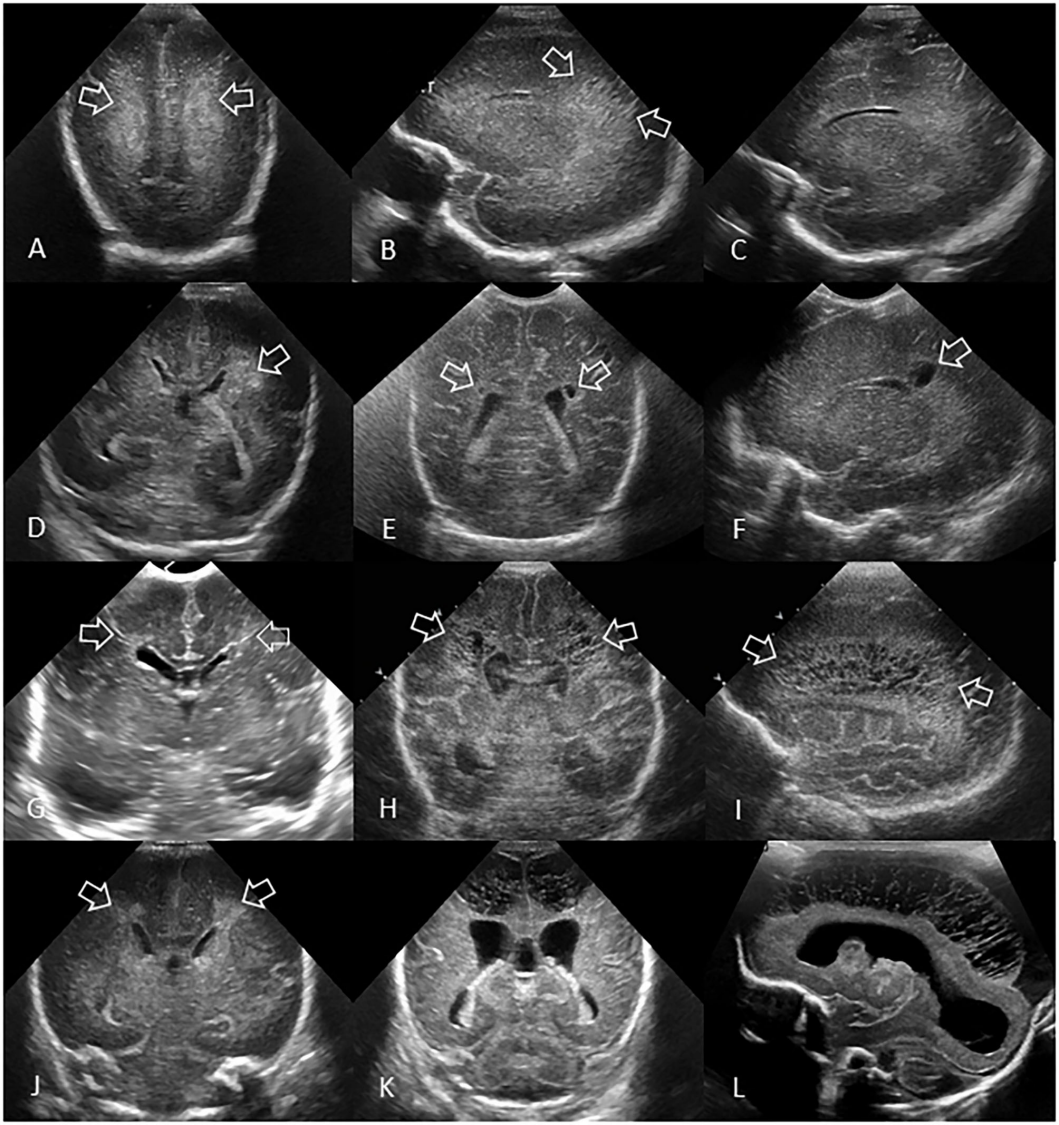
Spectrum of ischemic white matter injury. **(A–C)** Grade 1 WMI. Ultrasound scan in a preterm infant (GA 26 weeks) with transient abnormal white matter echodensities. **(A,B)** Coronal and left parasagittal scans show inhomogeneous moderately increased echogenicity (“flaring” or PVE) of the parietal periventricular white matter (arrows). No IVH. **(C)** Parasagittal scan 5 weeks later shows full resolution of the echodensities. White matter echogenicity is now homogeneous and normal, and no cysts developed. **(D–F)** Grade 2 WMI. Ultrasound scan in a preterm infant (GA 27 weeks). **(D)** Coronal scan at the level of the body of the lateral ventricles shows asymmetric inhomogeneous PVE (“flaring”) in the left parietal periventricular white matter (arrow). **(E,F)** Coronal and left parasagittal scans 9 weeks later showing small localized cystic lesions in the fronto-parietal white matter of both hemispheres (arrows). The tiny, right-sided cysts developed despite any convincing increased white matter echogenicity detected on early cUS exams. **(G–I)** Grade 3 WMI. Ultrasound scan in a preterm infant (GA 30 weeks with giant omphalocele partially repaired at day 4 of birth) with significant WMI. **(G)** Coronal scan 3 days after birth, at the level of the frontal horns of the lateral ventricles, shows only limited patchy areas of echodensity (arrows) not with certainty distinguishable from physiologic frontal echogenic areas in the periventricular white matter. No IVH. **(H,I)** Coronal and left parasagittal scans 4 weeks later show evolution of the WMI with extensive cystic lesions extending from the frontal white matter to the posterior parietal white matter (arrows) not expected by the only limited patchy echogenicity seen on day 3. The subcortical white matter is not involved. Dilated ventricles are passive in origin and resulting from tissue volume loss. **(J–L)** Grade 4 WMI. Ultrasound scan in a preterm infant (GA 25 weeks) with significant WMI. **(J)** Coronal scan 5 days after birth, at the level of the body of the lateral ventricles, shows patchy areas of echodensity (arrows) in the periventricular frontoparietal white matter. No IVH. **(K,L)** Coronal and right parasagittal scans 3 weeks later show evolution of the WMI with extensive cystic lesions extending from the frontal to the posterior parietal white matter. The progression of WMI could be recognized thanks to serial cUS exams as its severity was not expected by the limited patchy echodensity originally seen on day 5. Dilated lateral ventricles are passive in origin and result from tissue volume loss.

Preterm ischemic white matter injury is typically diagnosed by the identification of bilateral, often ill-defined and/or inhomogeneous areas of increased echogenicity in the periventricular white matter (Figure 7). Often, the echogenicity of the choroid plexus is used as a reference: if the echogenicity of (areas within) the periventricular white matter is increased as compared to the echogenicity of the choroid plexus, this is considered abnormal and a sign of white matter injury. Mild, bilateral, better defined echogenic areas within the white matter (especially peritrigonal “blushes” and symmetric frontal echodensities) are normal phenomena, often seen in very preterm infants (30).

### Differentiation of PVHI and Acute White Matter Injury

It is important to differentiate between hemorrhagic and non-hemorrhagic ischemic white matter injury as the prognoses and required early interventions may differ (8, 31). Non-hemorrhagic white matter injury, which in some cases leads to cystic white matter injury (see section White Matter Injury), is typically but not necessarily bilateral and ill-defined. In contrast, PVHI is more commonly “fan-shaped”, unilateral and associated with an ipsilateral IVH (28). The first key question to help make this distinction will be “Is there evidence of blood or a clot in or around the germinal matrix or in the lateral ventricles?” If blood is present at the side of the white matter lesion, and the lesion is asymmetric or unilateral, the lesion is considered to be of hemorrhagic infarction nature and labeled as PVHI. If bilateral areas of white matter echogenicity brighter than the choroid plexus are present in the absence of blood in the germinal matrix or ventricles, or are co-existing with only a small GMH, the white matter lesions are more likely to be of primary ischemic nature (Table 1). Both ischemic white matter injury and PVHI require follow-up cUS imaging as they increase the risk of cystic white matter injury or porencephalic cyst, respectively.

## Ventricular Measurements

Ventricular dilatation can be quantified with measurement of lateral ventricular size. Various parameters, such as the ventricular index (VI), anterior horn width (AHW), and frontal and temporal horn ratio (FTHR), are used to measure the ventricles. Reference values for these measurements have been described previously (32, 33).

### Reference Point

**Figure 8:**
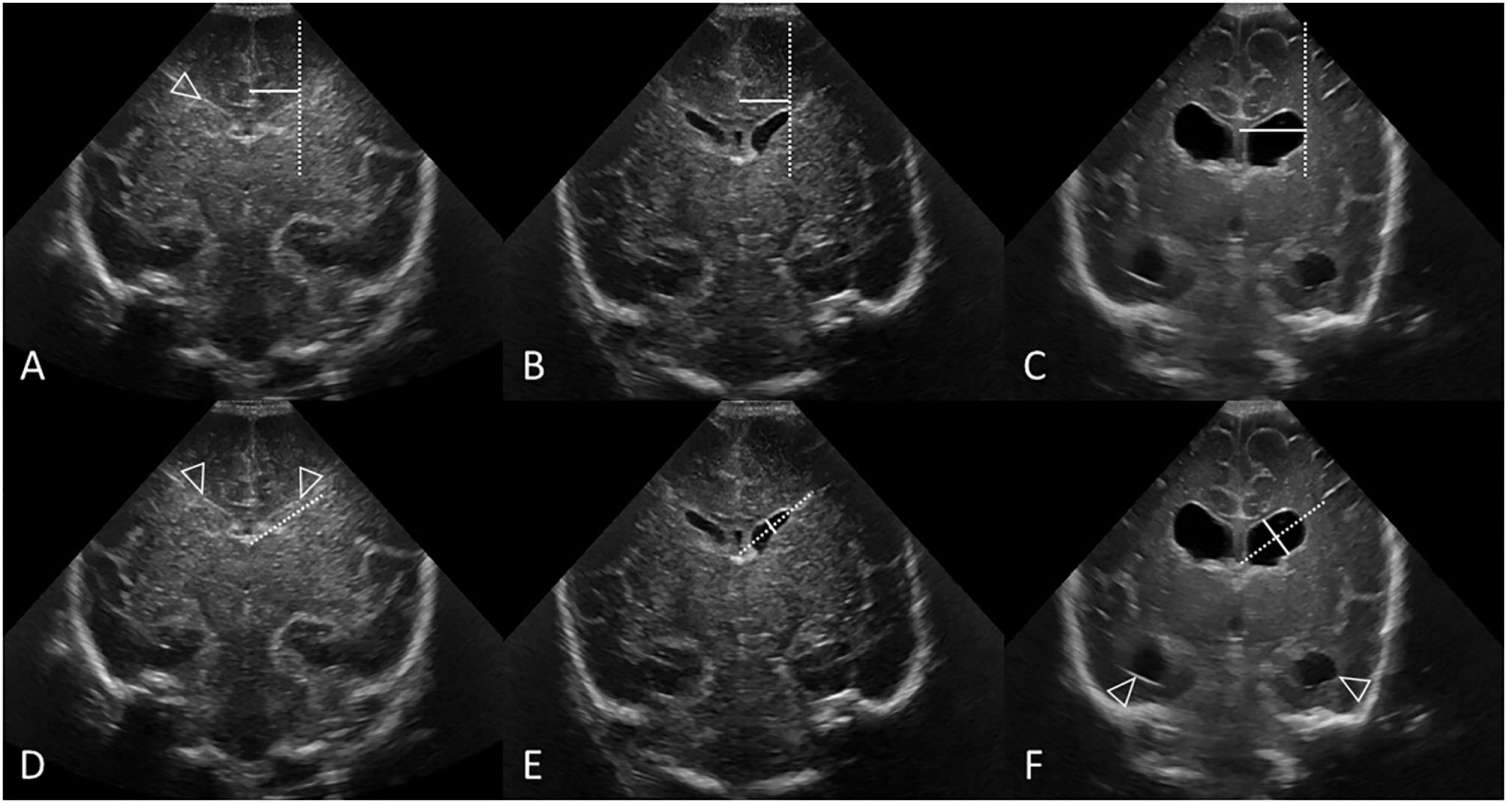
Measuring VI **(A–C)** and AHW **(D–F)** with clear ventricular borders. Coronal ultrasound scans in three preterm infants at the level of the frontal horns of the lateral ventricles. **(A–C)** Ventricular Index (VI) measurements (solid line) and reference aid (dotted line). The frontal horns are measured at the level of the choroid plexus as it resides in the third ventricle, also the level of the foramen of Monro. **(A,D)** Normal cUS. In **(D)**, the reference aid (dotted line) is placed inferior to the actual frontal horn for illustration purposes. The lateral ventricular margin can be identified by its thin echogenic ependymal stripe (arrowheads) which is helpful to measure VI when ventricular reopening has not yet occurred. **(B,E)** Normal cUS. The ventricular margins are now easily identified for measurement when the ventricles become mildly distended with CSF. Normal thin uniform echogenic ependymal stripe. **(C,F)** Post-hemorrhagic ventricular dilatation in late stage after resolution of the clot. Note also the distended temporal horns of the lateral ventricles (arrowheads).

**Figure 9:**
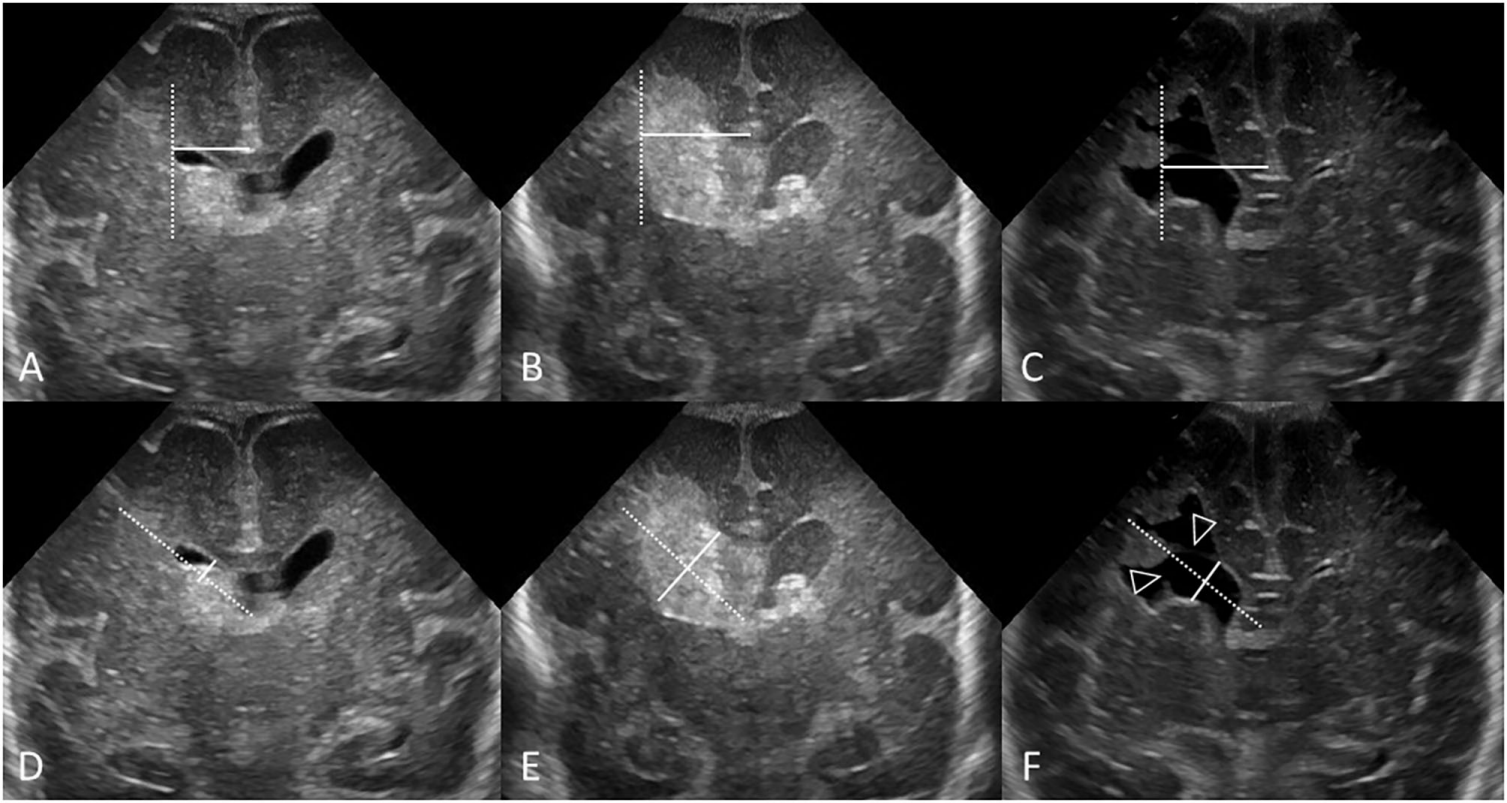
Measuring VI **(A–C)** and AHW **(D–F)** with obscured ventricular borders. Coronal ultrasound scans in 3 different preterm infants at the level of the frontal horns of the lateral ventricles. **(A–C)** VI measurements (solid line) and reference aid (dotted line). **(D–F)** Anterior horn width measurement (solid line) and reference aid (dotted line). It can occasionally be challenging to measure ventricular size when the ependymal margin of the ventricle is either obscured by echogenic clot **(B,E)** or interrupted by porencephalic cyst **(C,F)**. **(A,D)** Grade I GMH-IVH. Small clot at the caudothalamic notch causes local extrinsic mass effect, “pushes” into, and partially effaces the frontal horn of the lateral ventricle. In cases like this, the AHW measurement should extend partially across the germinal matrix clot to reach the imaginary continuation of the lower ventricular wall as its reference point. **(B,E)** Grade III GMH-IVH with frontal PVHI. In this example, the correctly measured AHW will extend partially across the echogenic clot, but not include the portion that is believed intraparenchymal in location. **(C,F)** Porencephalic cyst. A ghost outline of the ventricle can occasionally still be seen (arrowheads) and aids definition of ventricular size. In this example, the correctly positioned reference aid across the long axis of the ventricle will assist measurement of the AHW (placed perpendicular to the reference aid).

The first step in the measurements is to identify consistent reference locations of measurement of the ventricles (Figures 8, 9). Using the anterior fontanelle as an acoustic window, the VI and AHW of both lateral ventricles should be measured on cUS in the coronal plane at the level of the foramen of Monro (which is also known as the third coronal plane) or of the third ventricle. Normal choroid plexus resides in the roof of the third ventricle, and its extension into the lateral ventricles through the foramen of Monro can be visualized as a midline transverse echogenic band on coronal views and will aid in determining the designated plane(s) for measurement purposes. This is the correct coronal location to measure VI and AHW, and is close to the plane where the cerebellum starts to appear in the coronal image when scanning from the frontal region toward the occipital region. This is the plane where the Sylvian fissures are Y-shaped and the hippocampal fissures are clearly and symmetrical round-shaped (Figure 3).

### Measurement Method

The second step is to define a consistent method of measuring the ventricles (Figures 8, 9). VI is the horizontal distance between the midline of the interhemispheric fissure and the most lateral wall of the anterior horn in the coronal cUS plane (measured at the level of the foramen of Monro). The VI is measured separately for the left and right lateral ventricle. The AHW is the maximal diagonal width of the anterior horn measured at its widest point in the same coronal cUS plane (at the level of the foramen of Monro, see section Reference Point). It is also measured for both lateral ventricles. Larger clot volumes that fill and distend the ventricle may obscure definition of the ependymal lining. The visual recognition of the ventricle boundaries (i.e., its ependymal lining) is often aided by scrolling back and forth through the image series. Without scrolling, feature detection of the ventricular margin may fade when examining only a single image in a static manner. One pitfall is that, in case of a porencephalic cyst, the enlarged ventricular size reflects adjacent tissue atrophy rather than increased pressure in the ventricular system. Attempts should be made to exclude the cyst from the measurement and use the (imagined) continuation of the ventricular wall as reference point for the measurement (Figure 9). Markup calipers on imaging workstations do not always permit precise pixel placement of the measuring tool, and some user judgement about what measurement seems reasonable may be required.

The most accurate and reliable measurement should be taken for the final assessment. When both sides and measurements are accurate then the worse (largest) measurement should be used to determine brain injury severity.

## Sequelae of Acute Brain Injury

### Defining PHVD

**Figure 10:**
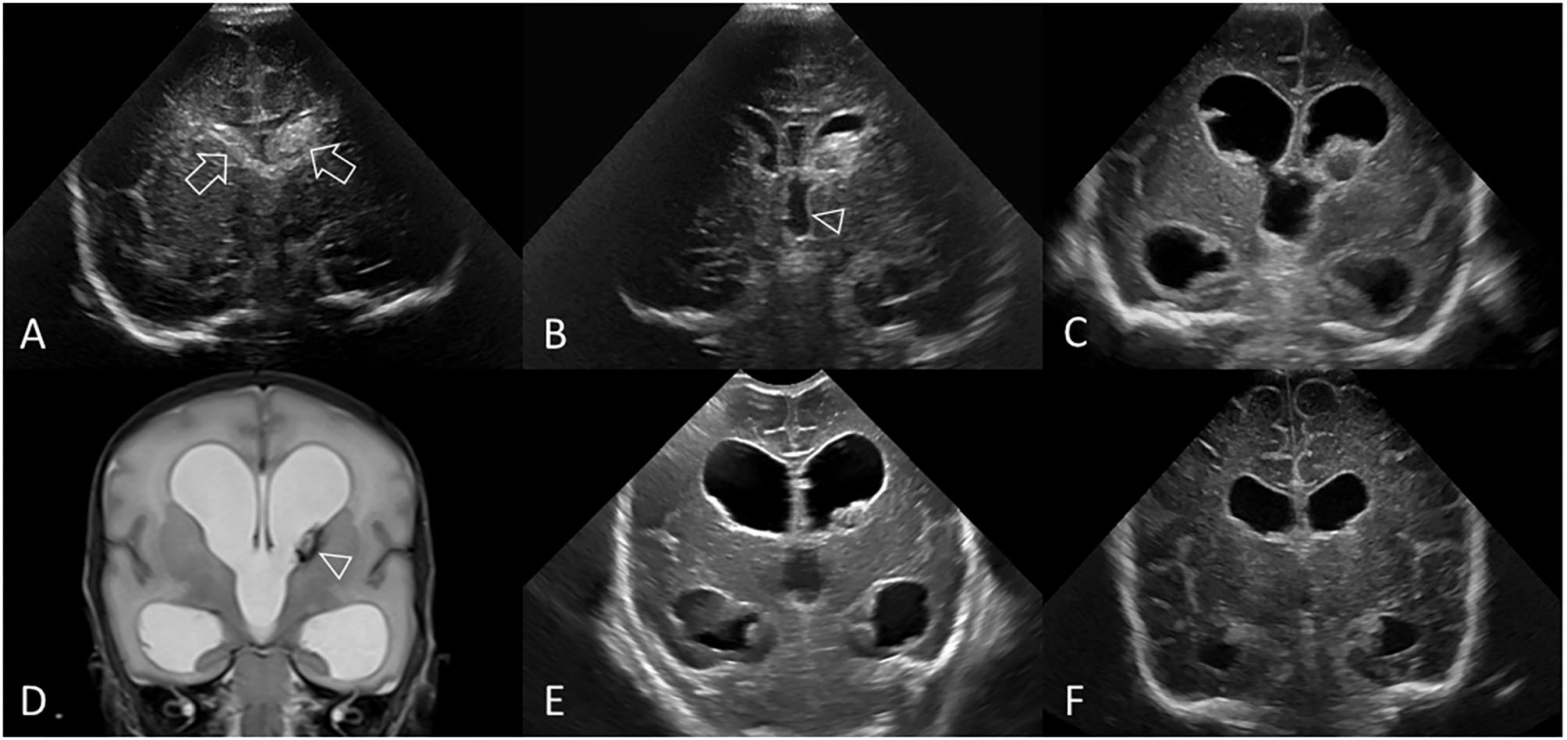
Post-hemorrhagic ventricular dilatation. Ultrasound scan in a preterm infant (GA 30 weeks) showing bilateral GMH-IVH. **(A)** Coronal scan 3 days after birth shows the bilateral hemorrhages (arrows). No PVHI. **(B)** Coronal scan at 8 days shows both lateral ventricles are beginning to dilate and also shows dilatation of the third ventricle (arrowhead). Right AHW <3 mm and left AHW = 4 mm (measurements not shown). **(C)** Coronal scan at 2 weeks shows ballooning of the lateral ventricles with bilateral AHW >6 mm (measurements not shown) and development of echogenic ependyma. Early clot regression. **(D)** Coronal T2-weighted MR image at 3 weeks shows persistent PHVD and small hypointense lesion at left caudothalamic notch representing hemosiderin staining (arrowhead). Note the absence of extracerebral CSF spaces. No PVHI. **(E)** Coronal scan at 4 weeks shows unchanged size of both dilated lateral ventricular bodies and temporal horns. Further regression of residual intraventricular clot. **(F)** Coronal scan at 10 weeks demonstrates stabilization of the PHVD with some decrease in ventricular caliber. The third ventricle is no longer dilated. Right and left AHW = 10 mm (measurements not shown). However, without dilated subarachnoid spaces or PVHI, the persistent enlarged lateral ventricles indicate continued presence of PHVD rather than passive enlargement from tissue volume loss. No surgical intervention. In this example, the frontal horn ballooning and largest size of the lateral ventricles was measured on the 2 week cUS exam (right AHW = 17 mm and left AHW = 15 mm, panel **(C)**, measurements not shown).

The third step is to define significant PHVD (Figure 10). PHVD remains an important complication of IVH in preterm infants and occurs in 30% to 50% of infants with GMH-IVH grades III or PVHI. Serial cUS is reliable for identifying and following the progression of PHVD and for aiding decision-making regarding the need for intervention. It is always crucial to perform a complete and comprehensive study of the ventricular system especially in case of a very large third ventricle, it is important to assess for an enlarged fourth ventricle in the midsagittal plane. In case of a tetraventricular dilatation (dilatation of the lateral ventricles, third and fourth ventricle), there is probably a fourth ventricular outflow obstruction. PHVD is to be graded based on measurement of the largest lateral ventricle after the first week of age. It is defined as moderate when VI >97^th^ centile and AHW > 6mm and severe when VI >97^th^ centile + 4 mm or AHW >10 mm. Bilateral VI and AHW measurements on each cUS exam are to be recorded. These measurements should be repeated on a regular basis in order to monitor PHVD and determine whether intervention is indicated.

VI and AHW graphs can be used to divide PHVD into three Neurodevelopmental outcome risk groups: low, moderate, and high (29). Electronic spreadsheets for these risk zones are available for postmenstrual ages 24 weeks to 42 weeks (https://tinyurl.com/PHVD-Measures-1) and for postmenstrual ages 24 weeks to 29 weeks (https://tinyurl.com/PHVD-Measures-2) (34).

### Other Methods of Assessing the Severity of PHVD

Another measurement that can be used to assess the severity of PHVD is the resistive index (RI) of the pericallosal artery. RI measurements have been used in the setting of preterm infants with PHVD to determine whether decompression of the ventricular system with an external drain should be performed (35, 36). The RI of the subcallosal artery can be calculated on serial cUS exams. A value of >0.85 suggests a low diastolic flow that occurs when cerebral swelling causes intracranial pressures to exceed systemic pressures; this, in turn, will impede antegrade or cranial-directed flow during diastole. An elevated RI will come down as PHVD improves, or after cerebrospinal fluid drainage through a ventricular access device or shunt. In the most severe cases, diastolic flow will be absent or reversed. However, obtaining consistent and reliable RI measurements is highly operator-dependent and is neither widely available nor part of most routine cUS exams. Thus, due to concerns about untested reproducibility, accuracy, reliability, and validity, obtaining RI measurements is not recommended in our consensus viewpoint at this time.

### Cystic White Matter Lesions

The periventricular white matter in preterm infants is immature, with migrating cells, developing tracts and abundant pre-myelinating oligodendroglia. Consequently, the white matter is vulnerable to injury, predominantly due to ischemia, infarction and inflammation. The injured tissue can become necrotic and subsequently cystic. Two main types of cystic white matter lesions can be distinguished: hemorrhagic (in the presence of IVH, often eventually leading to porencephalic cysts) and ischemic (in the absence of GMH-IVH), both of which can have major implications for neurodevelopmental outcomes (9). Cysts due to ischemic white matter are usually identified beyond 2 to 4 weeks after birth but can still develop at any point over the course of the neonatal period, as ischemic white matter injury can develop several weeks after birth following an acute illness such as sepsis or necrotizing enterocolitis (37).

### Porencephalic Cyst

In the setting of neonatal brain injury, a porencephalic cyst is a cystic lesion that may develop in the same location as a previous PVHI following coagulative necrosis of the infarcted tissue. It generally develops within several weeks from the onset of PVHI and becomes increasingly echolucent and CSF-filled over time. The cavity will usually, but not always, communicate with the adjacent ventricle (Figure 8). For the purpose of this article, the term ‘porencephalic cyst’ will have this intended meaning. This definition is different from that used in the radiology literature, where ‘porencephalic cyst’ has a broader meaning and may be used to indicate a CSF-filled parenchymal cavity (congenital or acquired) that typically communicates with the ventricular system.

**Figure 11:**
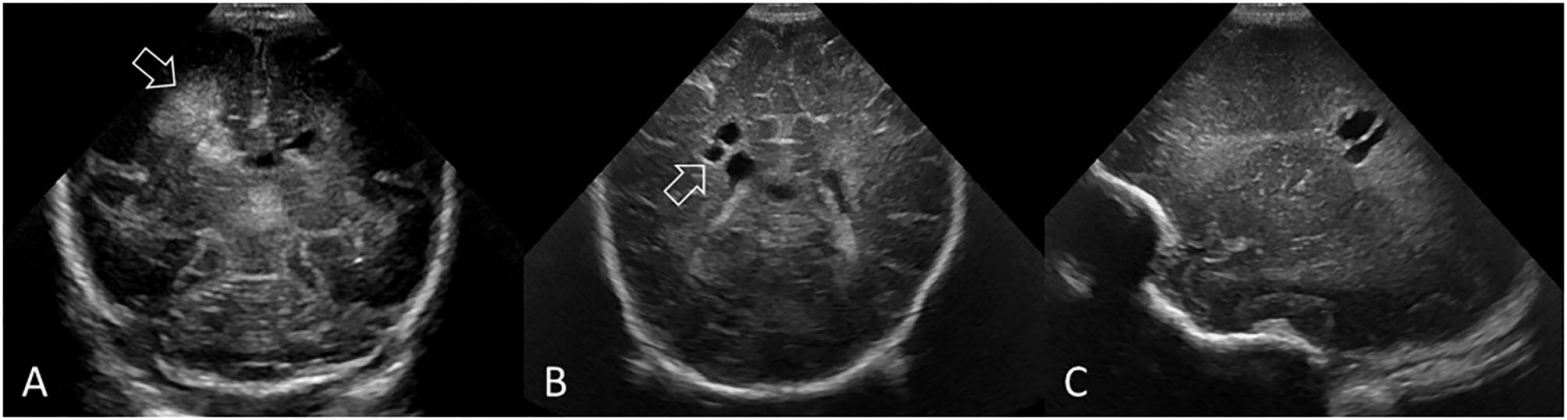
PVHI with eventual evolution into multiple, unilateral cystic lesions separate from the lateral ventricle in a preterm infant (GA 25 weeks). **(A)** Coronal ultrasound scan 5 days after birth showing the right-sided GMH-IVH and large echodensity in the periventricular white matter adjacent to, but not communicating with, the lateral ventricle (arrow). **(B,C)** Coronal and right parasagittal scans 11 weeks later show cystic lesions in the parietal white matter that do not communicate with the lateral ventricle (arrow). PVHI adjacent to, but not communicating with, the lateral ventricle often evolves into multiple smaller cystic lesions rather than one large porencephalic cyst. This small cluster of white matter cysts could be mistaken for grade 2 ischemic WMI. However, review of the baseline cUS exam and understanding the evolution of injury clarifies that this represents variant cystic evolution of PVHI.

A porencephalic cyst needs to be differentiated from a grouped collection of multiple, mostly smaller parenchymal cysts (each usually 2-5 mm in diameter). PVHI that occurs separate from the lateral ventricle can result in multiple cysts separate from the lateral ventricle and be mistaken for unilateral cystic white matter injury. The visual identification of clot evolution on serial cUS should help avoid this interpretative error in most cases (Figure 11). Another important point to aid differentiation is that ischemic cystic white matter injury is more typically bilateral in location.

### Non-cystic White Matter Injury

Ischemic white matter injury (WMI) is often initially visible on cUS as abnormal echogenicity of the periventricular white matter. The term ‘periventricular leukomalacia’ (PVL) was previously used to describe this pattern of ischemic injury, but ischemic WMI is now more commonly labeled simply as “WMI.” More recently, cystic WMI (c-PVL) has become rare and non-cystic WMI has become the predominant form of WMI, probably occurring in more than 50% of the very preterm population (18). Although non-cystic WMI is not always reliably detected by cUS, early cUS may show increased and inhomogeneous periventricular echodensities. Persistent white matter echogenicity can over a period of time (weeks) lead to white matter volume loss, which can be seen on cUS as irregular dilatation of the lateral ventricles, widening of the interhemispheric fissure and extracerebral spaces, and plump, wide gyri. Unless they are inhomogeneous, areas of increased echogenicity that resolve within the first week of birth are generally not considered pathologic and probably caused by transient venous congestion (14).

The reference for abnormal periventricular echogenicity (PVE) is the echogenicity of the choroid plexus. If the echogenicity of the abnormal white matter exceeds that of the choroid plexus (brighter) or is inhomogeneous, then it should be labeled as PVE. Subtle, homogeneous echogenicity within the white matter that does not exceed the echogenicity of the choroid plexus can be considered physiologic (30).

WMI can be classified based on the occurrence, progression over time, and extent of lesions (Figure 7), as previously described (38).

Grade 1: Transient areas of increased PVE persisting for ≥7 days (Figures 7A–C).
Grade 2: Transient PVE that evolves into small, localized cysts in the fronto-parietal periventricular white matter (Figures 7D–F).
Grade 3: PVE evolving into extensive periventricular cystic lesions in the fronto-parieto-occipital white matter (Figures 7G–I).
Grade 4: PVE evolving into extensive cystic lesions in the deep white matter or in the subcortical white matter (Figures 7J–L).

### Less Common Forms of Preterm Brain Injury

**Figure 12:**
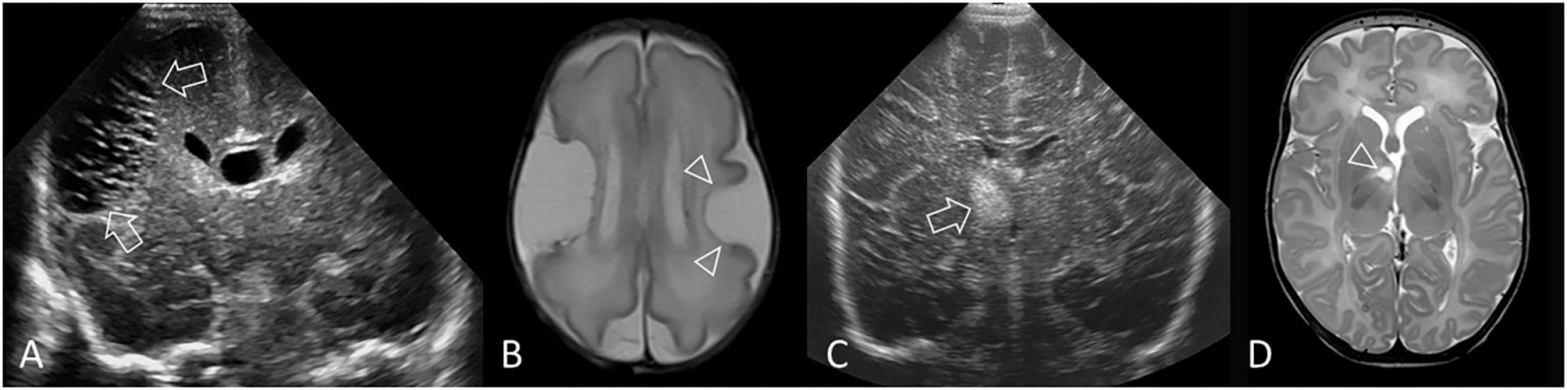
Other forms of acute injury. **(A,B)** Preterm infant (GA 27 weeks) with bilateral middle cerebral artery (MCA) territory infarction. **(A)** Coronal ultrasound scan 3 weeks after birth shows extensive cystic injury in the right frontoparietal region (arrows), but located more lateral than expected for common periventricular WMI. Focal echodensity was first identified at this location on cUS 7 days after birth (not shown). **(B)** Axial T2-weighted MR image shows the large right MCA cortical infarct and an additional smaller left MCA cortical infarct (arrowheads) that was not readily appreciated on cUS because of its far lateral location. The bilateral MCA strokes suggest a proximal embolic source, and placenta was suspected. **(C,D)** Perforator stroke in a preterm infant (GA 34 weeks). **(C)** Coronal ultrasound scan, at the level of the third ventricle and foramen of Monro, performed after surgery for ventriculoseptal defect closure shows a focal echodensity in the medial right capsuloganglionic region (arrow). cUS performed prior to surgery did not show any abnormality. Findings are most consistent with lenticulostriate territory infarction (middle cerebral artery perforating branches). **(D)** Axial T2-weighted MR image after 2 weeks shows cystic evolution of the infarct without evidence of previous hemorrhage (arrowhead).

In acute embolic cerebral infarction, cysts will also develop over the course of 2-3 weeks of the onset of infarction. These cysts are mostly unilateral and occur within an arterial territory, and therefore easy to distinguish from c-PVL (Figures 12A,B). Also cortical strokes commonly occur in different sonographic locations. Isolated perforator infarcts are another relatively common cause of perinatal stroke in preterm infants and are often asymptomatic and discovered incidentally on imaging exams in the first weeks after birth (39, 40). Perforator strokes can be seen as well-delineated hyperechoic lesions in the striatum or thalamus on cUS (Figures 12C,D). Lenticulostriate (perforator) infarcts are typically wedge-shaped and are commonly encountered in the preterm population. They are typically seen beyond the first week after birth.

## Definition of Significant Preterm Brain Injury

We propose defining significant or severe preterm brain injury when any of the following is present: IVH grade III or PHVI, severe ventricular dilatation, cystic white matter lesions, and/or moderate to large CBH. These lesions are known to be associated with ND problems later in life.

## Timing of cUS Exams

**Figure 13:**
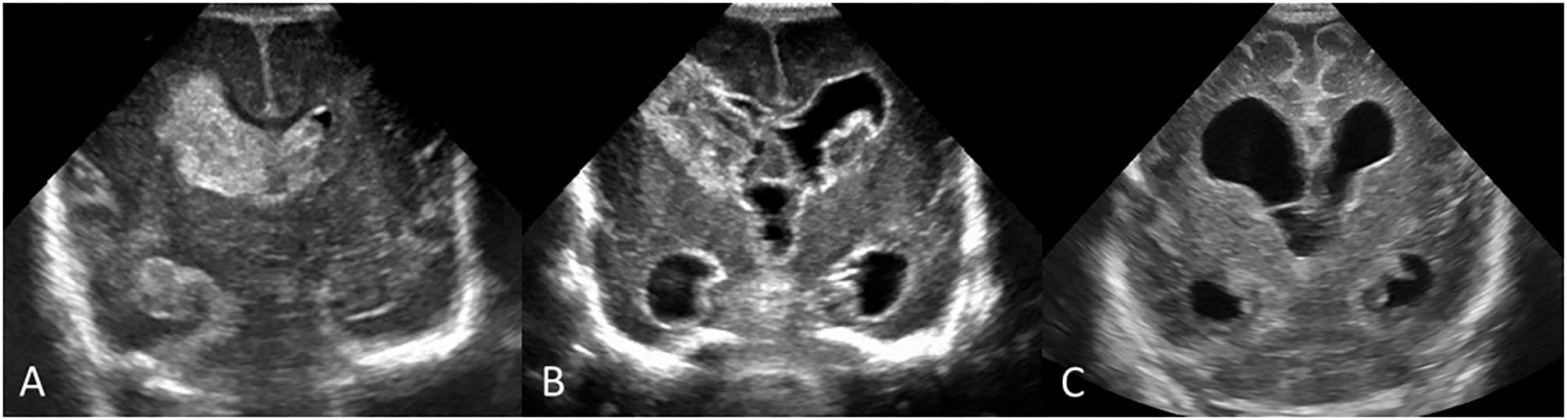
Early and follow up serial cUS scans showing evolution of an acute brain injury in a preterm infant (GA 24 weeks) with bilateral IVH. **(A)** Coronal scan, at the level of the frontal horns of the lateral ventricles, shows a large acute right-sided GMH-IVH with PVHI and smaller left GMH-IVH. **(B)** Follow-up coronal scan 2.5 weeks after birth shows IVH evolution with organizing clot and the development of PHVD; note enlarged third ventricle and dilated temporal horns. Hyperechogenic ependyma has developed. **(C)** Follow-up coronal scan at term-equivalent age shows end-stage pattern effects of earlier injuries with mature right periventricular porencephalic cyst and development of some diffuse cerebral atrophy depicted by widening of convexity cortical sulci and interhemispheric fissure. PHVD has improved, with residual chronic ventriculomegaly likely reflecting a combination of passive enlargement from brain atrophy and impaired CSF resorption pathways from earlier intraventricular blood.

**Figure 14:**
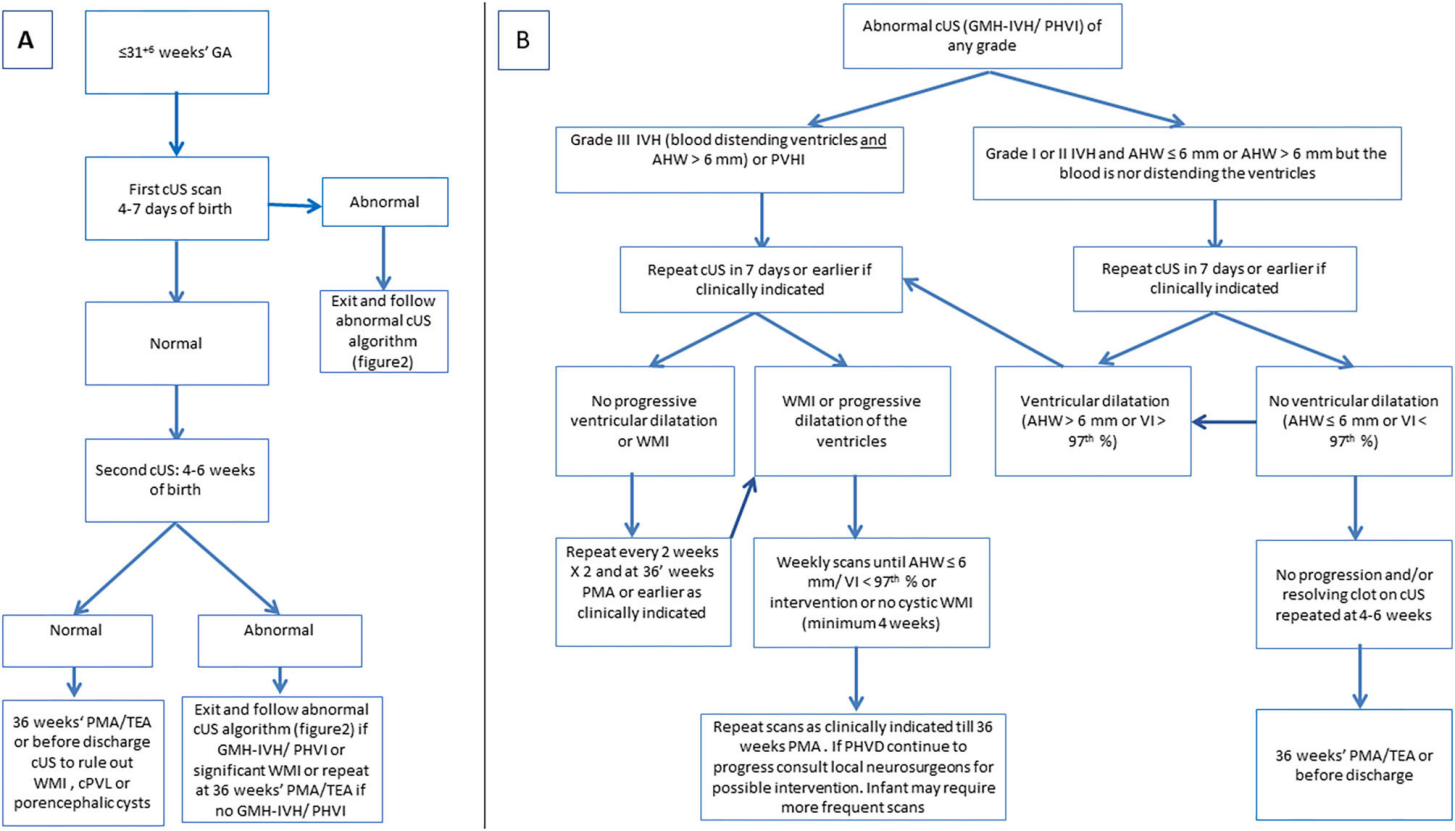
**(A)** Algorithm for routine cranial ultrasound screening for infants born at or equal to 31 weeks' gestation. **(B)** Algorithm for cUS scenario of abnormal cUS findings.

Serial cUS is required to identify and document the full spectrum of acquired brain injury in very preterm infants (Figure 13). Currently, recommendations on the timing of cUS assessments in preterm neonates are varied. A consensus approach to timing of cUS was developed for the Canadian situation. This approach recommends performing at least three sequential routine screening cUS studies during the neonatal period up to 36 weeks' postmenstrual age or term equivalent age (TEA), with the first cUS between day 4 to 7 after birth, second cUS between 4 and 6 weeks after birth, and third cUS at approximately 36 weeks' corrected GA (Figure 14A). This cUS schedule should be intensified in cases of detected abnormalities or when clinically indicated (Figure 14B). It should be realized that European groups generally advise more intensive and frequent cUS examinations (15). We strive to more consistency between North-American and European recommendations in the future, based on intercontinental multi-center studies, assessing the diagnostic and prognostic accuracy of frequent vs. less frequent cUS examinations.

cUS within the first day after birth will allow for identification of antenatally acquired injury and congenital malformations. In a stable infant, without suspicion of antenatal injury, the first cUS examination may be postponed to day 4 to 7 after birth, thus avoiding scanning within the first 72 hours of birth. This 72-hour window is regarded as the critical window in which a focus on nurturing care with minimal handling and intervention has been shown beneficial to decrease the risk of preterm brain injury (39, 41).

The rationale for the proposed timeline for screening cUS was as follows: The cUS during the first week is intended to detect GMH-IVH, PVHI, early onset PHVD, and CBH; the cUS performed after 4-6 weeks will help identify the evolution of GMH-IVH or uncommon late-onset IVH and CBH, significant white matter injury and focal arterial infarction; and scanning again near TEA will provide an assessment of how the brain has matured and will more fully characterize any permanent effects of earlier brain injury (40) (CEBM Level of Evidence 1).

In infants with hemorrhagic injury (GMH-IVH and/or CBH), cUS needs to be intensified for timely detection of PHVD, allowing timely intervention (34).

Outside the proposed timeline, circumstances where cUS screening should be considered include: infants with risk factors such as a 5-minute Apgar score of <7, rapidly falling hemoglobin level, clinical deterioration with hemodynamic instability (hypotension requiring intervention, shock, or hemodynamically significant PDA), fulminant NEC or septic shock (positive body fluid culture and hypotension), or requirement of mechanical ventilation on day of birth (42).

## Discussion

In this Canada-wide initiative, we aimed to define preterm brain injury, produce a consensus document outlining limitations of the current approach and providing guidelines for conducting cUS examinations. Prevention of preterm brain injury has been the goal of multiple research studies and quality improvement initiatives worldwide. However, comparing different practices to identify those associated with reductions in preterm brain injury is complicated by variability in the definitions, imaging protocols, and classification systems used. The first step in addressing this variation is standardization of the outcome measurement, which is a cornerstone in any quality improvement project (43). Without clear outcome definition and accurate measurement tools, assessing interventions becomes very challenging and can create false reassurance or frustration (44).

The ideal brain injury outcome measure should be easily applicable, widely communicated, and supported by data (45). As a simple, reliable (highly sensitive), and safe bedside test that can be repeated as indicated, cUS is the modality of choice for evaluating preterm brain injury (40).

Several existing classification systems provide guidance on grading of preterm hemorrhagic and ischemic brain lesions as detected on cUS in preterm infants (3, 18, 30). However, a comprehensive classification of preterm brain injury using cUS, which includes recommendations on timing of the routine assessment and follow up for hemorrhagic and ischemic lesions, has been rarely reported. Our definition of significant or severe brain injury for the purpose of Quality improvement of the Canadian Neonatal Network reporting (the presence of any of: IVH grade III or PHVI, severe ventricular dilatation, cystic white matter lesions, or moderate to large CBH) is in agreement with the reported literature (3, 8, 18, 23, 38). In addition to the known association of severe IVH with neurodevelopmental impairment in preterm children, cystic white matter lesions independently predict cerebral palsy and CBH is associated with cognitive and motor delay (46).

The Australian and New Zealand Neonatal Network studied the reliability of brain injury reporting using cUS as the imaging tool as a step toward standardization of preterm brain injury outcome definitions and measures. They identified similar sources of variation in brain injury definitions: acute preterm brain injury grading, ventricular dilatation assessment and timing and frequency of scans (47). Our proposed classification has five main advantages that overcome the limitations of previous systems: (1) standardization of timing and frequency of cUS scanning for brain screening and injury diagnosis, (2) clear differentiation between ischemic and hemorrhagic WMI, (3) emphasis on the significance of cerebellar injury in addition to other ischemic or hemorrhagic brain lesions, (4) clear differentiation between acute ventricular dilatation related to grade III IVH from PHVD, and (5) standardization of the definition of PHVD based on plotting lateral ventricular measurements on validated charts (34).

The proposed cUS protocol is consistent with the recent Canadian Pediatric Society position statement (2020) in relation to the population targeted (routinely for preterm infants born before 32 weeks' gestation or those with hemodynamic instability or critical illness requiring vasopressors, sepsis, or NEC); the technique of cUS acquisition (anterior and mastoid fontanelles); the timing of routine diagnostic scans (during the first week and at 4-6 weeks of chronological age); and the need for follow-up cUS if abnormality is detected on the initial scans (48). However, the algorithm we propose in this article addresses an area identified as a source of between-center variability in cUS imaging by standardizing the timing of follow-up after an initial abnormal cUS scan. The proposed algorithm also emphasizes the importance of sequential cUS including a TEA or near TEA scan as diagnostic and prognostic tool especially when utilizing a combination of markers such as ventricular size, subarachnoid space, frontal inter-hemispheric fissure distance, corpus callosum thickness, cerebellar injury, and cortical maturation (49). Furthermore, sequential cUS up to term or near-term age has been shown to improve the sensitivity and the long-term neurodevelopment predictive ability of cUS (10, 50).

Our proposed protocol mitigates the limitations of previous protocols in relation to the frequency of cUS imaging in preterm infants. Two extreme approaches exist in the literature, ranging from weekly scans to as few as two scans from birth until TEA (50–52). The former practice has the advantage of better identification of subtle WMI that may be missed or have “pseudo-normalized” with fewer scans, thus decreasing false negative results. However, it has also raised concerns about parental anxiety, cost, and a lack of therapeutic interventions in case of abnormal imaging findings. Our protocol overcomes the limitation of few scans proposed by the recent position statement from the Canadian Pediatric Society while supporting sequential cUS in an attempt to improve the sensitivity and long term neurodevelopment outcome predictive accuracy of cUS.

## Strengths

Strengths of our work include the provision of a classification system for brain injury related to prematurity, standardization of a cUS imaging algorithm, and the evidence-based approach. The proposed classification of preterm brain injury encompasses GMH-IVH, significant ventricular dilatation, cerebellar injury, and white matter ischemic and hemorrhagic lesions – all of which are independent predictors of long-term neurodevelopmental outcome. We also propose sequential cUS, including routine term or near-term age scans to assess brain growth and maturation. Additionally, this initiative provides a framework for ongoing research and quality improvement projects aiming to identify the best practices associated with improved outcomes for preterm children and their families.

The major strength of our work is in the method we used to generate our consensus classification system and algorithm. More specifically, we brought together experts in neonatology and radiology representing all 31 tertiary NICUs in Canada and conducted multiple interactive initiatives, including surveys, hands-on cUS workshops using simulations and phantoms, small groups discussions, and polling cUS interpretation through questionnaires. Through this process, we clearly revealed the points of variation among the centers in their approaches to cUS imaging and classification. We also increased engagement and buy-in from stakeholders and frontline healthcare providers, which in turn will increase our chances of successful implementation. The proposed consensus algorithm provides standardized timing and frequency of follow up for cUS scanning that may balance cost effectiveness.

## Limitations

The main limitations of this initiative are essentially those of the imaging tool used. It is known that cUS is less sensitive than MRI for the detection of predominantly small and subtle forms of neonatal brain injury and those located in the posterior fossa and periphery of the brain (53). However, our proposed sequential cUS algorithm is designed to improve the sensitivity and predictive value of cUS, in an attempt to limit the harm associated with relaying false positive or false negative results to care providers or families. Furthermore, the inter-observer reliability of cUS is generally poor for low-grade IVH and mild parenchymal echogenicity (47, 54). Due to differences in available resources and expertise at local sites, care providers should be aware of inter-observer differences in the interpretation of cUS regardless of the classification system used. Less frequent cUS may increase the risk of missing WMI or window of intervention for PHVD. Another limitation of the study is the use of AHW and a cut-off of 6 mm to diagnose grade 3 IVH which may create confusion with the method used for PHVD assessment. Finally, we did not perform a cost-benefit analysis to assess whether the proposed cUS algorithm would result in improved identification of preterm brain injury at the cost of an increased number of cUS scans or referrals for early interventions.

## Evaluation plan for the proposed classification

The proposed consensus classification and cUS protocol for preterm brain injury have been evaluated as follows:

- Formative evaluation was conducted to ensure that the proposed cUS protocol is feasible, appropriate, and acceptable to all stakeholders. This was done via surveys, small groups discussions, and two large group meetings. Stakeholders included neonatologists, radiologists/neuroradiologists, nurses, families, and administrators. The feedback received from these stakeholders was integrated to modify the draft versions of this protocol and to develop the final consensus document proposed. This evaluation was conducted prior to implementing this initiative to engage Canadian tertiary centers and to maximize the chances for success.

Future plans for evaluation include the following:

- Process/implementation evaluation to determine if this initiative is accessible and acceptable to the targeted groups (radiologists and neonatologists), to assess compliance with the proposed algorithm, and identify facilitators and barriers to its implementation. This information will be collected from surveys, CNN data, and group meetings of the Neonatal Neurological Outcomes Improvement Group.
- Outcome/effectiveness evaluation will measure the variability in preterm brain injury before and after implementation of this proposed cUS classification. The after-implementation measures will be collected once we are satisfied that the implementation process was achieved as intended.
- Comparison between North-American and European cUS guidelines with regards to diagnostic and prognostic accuracy

## Implications and Future Directions

Our group is planning to develop a national neonatal cUS training program to standardize cUS image acquisition, interpretation, and reporting for the very preterm population across Canadian tertiary centers. Once center variability in relation to cUS protocols and definitions of preterm brain injury has been reduced, we will begin comparative effectiveness research and work on revising and expanding the classification further to address important issues such as the extend and location of the PVHI and intraparenchymal hemorrhage (21, 55). These studies will enable comparison of evidence-based interventions targeting the prevention of preterm brain injury and will identify those centers with low rates of brain injury (adjusted for their population characteristics). Other modalities of cranial ultrasound might be explored as they become available and studied such as 3D ultrasonography and cerebral venous system Doppler to identify cerebral venous thrombosis.

Finally, we will carry out knowledge dissemination activities to promote learning based on those centers' best practices associated with the lowest rates of brain injury and the optimal long-term neurodevelopmental outcomes.

## Conclusions

Brain injury remains a common and clinically significant morbidity in very preterm infants and is associated with mortality and adverse neurodevelopmental outcomes. cUS is critical for timely diagnosis and classification, and to guide family counseling and management. This consensus viewpoint was developed collaboratively by Canadian neonatologists and radiologists to provide an overview of the types, characteristics, and complications of major preterm brain injury identified on cUS, and to provide a standardized approach for screening and follow-up. We believe that standardizing the approach for assessing preterm brain injury using cUS will decrease subjectivity in cUS assessment, improve detection of injury and prediction of outcomes, and facilitate targeted quality improvement initiatives for optimizing patient care.

## Data Availability Statement

The original contributions presented in the study are included in the article/Supplementary Material, further inquiries can be directed to the corresponding author/s.

## Author Contributions

KM led the project, designed the surveys, developed the simulators, conducted the workshops, and wrote the first draft of the manuscript. JS designed the 4-step approach to grading GMH-IVH, conducted the workshops, prepared all figures and figure legends, and wrote the first draft of the manuscript. LL, HZ, and JA conducted the workshops, reviewed the survey and reviewed the first and revised versions of manuscript. BP led the drive to zero IVH project, reviewed and approved the final manuscript. LV and GW-M reviewed the first and revised versions of the manuscript. SL sponsored the initiative reviewed and approved the final version of the manuscripts. PS sponsored the initiative, organized the workshops, reviewed the first and revised versions of the manuscripts. All authors contributed to the article and approved the submitted version.

## Conflict of Interest

The authors declare that the research was conducted in the absence of any commercial or financial relationships that could be construed as a potential conflict of interest.

## Abbreviations

AHW: Anterior horn width
CBH: Cerebellar hemorrhage
CEBM: Centre for Evidence-Based Medicine
CNN: Canadian Neonatal Network
CPTBN: Canadian Preterm Birth Network
CSF: Cerebrospinal fluid
cUS: Cranial ultrasound
EPIQ: Evidence-based Practice for Improving Quality
GA: Gestational age
GMH-IVH: Germinal matrix hemorrhage – Intraventricular hemorrhage
IVH: Intraventricular hemorrhage
MRI: Magnetic resonance imaging
NEC: Necrotizing enterocolitis
PVE: Periventricular echogenicity
PVHI: Periventricular hemorrhagic infarction
PVL: Periventricular leukomalacia
PHVD: Post-hemorrhagic ventricular dilatation
PMA: Postmenstrual age
TEA: Term equivalent age
VI: Ventricular index
WMI: White matter injury.
