# Supplementary material for: Consensus Approach for Standardizing the Screening and Classification of Preterm Brain Injury Diagnosed With Cranial Ultrasound: A Canadian Perspective
Source: Front Pediatr. 2021 Mar 8;9:618236. doi: 10.3389/fped.2021.618236 (PMC7982529; doi:10.3389/fped.2021.618236)
Supplement: Supplementary file 1 [file Table_1.DOCX]

| Steps for Classifying GMH-IVH in Preterm Infants |
| --- |
| STEP #1: Is there hemorrhage within/around the germinal matrix AND/OR within the lateral, third, or fourth ventricle?  NO: Report negative for GMH-IVH, go to Step #4.  YES: Report positive for GMH-IVH, go to Step #2.  STEP #2: Is the hemorrhage confined to the germinal matrix region?  NO: Blood is detected in the lateral ventricle or on the choroid plexus = Grade II or III GMH-IVH, go to Step #3.  YES: Either common (Image 3) or uncommon (Image 4) in location AND no blood in lateral ventricle = Grade I GMH-IVH, go to Step #4.  STEP #3: Is the IVH distending the ipsilateral lateral ventricle AND the AHW measures >6 mm?  NO: Grade II GMH-IVH (Images 5,6), go to Step #4.  YES: Grade III GMH-IVH (Image 7), go to Step #4.  STEP #4: Is focal echogenicity present in the periventricular white matter adjacent (i.e., ipsilateral) to the side of GMH-IVH or, in case of bilateral GMH-IVH, ipsilateral to the largest GMH-IVH?  NO: If negative for GMH-IVH in Step #1, report as normal.  NO: If positive for GMH-IVH in Step #1, report as defined in Steps #1-3.  YES: Is there Grade I, II, or III GMH-IVH present?   - - If no GMH-IVH present, report as ischemic injury (Image 8).   - If GMH-IVH present, report as PVHI (Image 9 and Table 1). |
