# Supplementary material for: Consensus Approach for Standardizing the Screening and Classification of Preterm Brain Injury Diagnosed With Cranial Ultrasound: A Canadian Perspective
Source: Front Pediatr. 2021 Mar 8;9:618236. doi: 10.3389/fped.2021.618236 (PMC7982529; doi:10.3389/fped.2021.618236)
Supplement: Supplementary file 2 [file Data_Sheet_1.PDF]

Supplementary Material.
Canadian Neonatal Network and Canadian Preterm Birth Network Group on Neonatal Neurological Outcomes Improvement Member Investigators

Name (Alphabetical)	Affiliation	Email
Dalal Abdelgadir	University of Alberta	dalal.abdelgadir@ahs.ca
Muzafar Gani Abdul Wahab	University of McMaster	abdulwmg@mcmaster.ca
Jehier Afifi	IWK health centre	Jehier.Afifi@iwk.nshealth.ca
Sajit Augustine	Windsor Regional Hospital	sajit.augustine@wrh.on.ca
Rudaina Banihani	Sunnybrook Health Sciences Centre	rudaina.banihani@sunnybrook.ca
Valerie Bertelle	University of Sherbrook	valerie.bertelle@usherbrooke.ca
Victoria Bizgu	Jewish General Hospital	victoriabizgu@gmail.com
Jayalakshmi Bodani	Regina General Hospital	Jaya.bodani@saskhealthauthority.ca
Michael Castaldo	University of British Columbia	michael.castaldo@cw.bc.ca
Anil Chacko	Surrey Memorial Hospital	dranilchacko@gmail.com
Natalie Chan	University of British Columbia	chan.natalie@gmail.comv
Paige Church	Sunnybrook Health Sciences Centre	Paige.Church@sunnybrook.ca
Kevin Coughin	Western University	Kevin.Coughlin@lhsc.on.ca
Orlando da Silva	London Health Sciences Centre	odasilva@uwo.ca
Christine Drolet	Universitaire de Quebec	drolet.christine@chudequebec.ca
Marc Elliott	Janeway Children's Health Centre	Marc.Elliott@easternhealth.ca

Amr El Shahed	The Hospital for Sick Children	amr.elshahed@sickkids.ca
Mansoor Farooqui	University of Manitoba	mfarooqui@hsc.mb.ca
Dilkash Kajal	Mount Sinai Hospital	dilkash.kajal@uhn.ca
Arlene Kanigan	University of Alberta	akanigan@ualberta.ca
Jaideep Kanungo	Victoria General Hospital	jaideep.kanungo@viha.ca
Edmond Kelly	Mount Sinai Hospital	Edmond.Kelly@sinaihealthsystem.ca
Bojan Kovacina	Jewish General Hospital	bojan.kovacina@mcgill.ca
Naeem Khan	IWK health centre	Naeem.Khan@iwk.nshealth.ca
Faiza Khurshid	Kingston Health Sciences Centre	faiza.Khurshid@kingstonhsc.ca
Shoo K. Lee	Mount Sinai Hospital	Shoo.Lee@sinaihealth.ca
Marie-Claude Lefebvre	University of Sherbrooke	mclefebvre@gmail.com
Lara Leijser	University of Calgary	Lara.Leijser@ahs.ca
Brigitte Lemyre	University of Ottawa	blemyre@toh.ca
Deepak Louis	St. Boniface General Hospital	Dlouis@hsc.mb.ca
Janette Mailo	University of Alberta	janette.mailo@ahs.ca
Edith Massé	University of Sherbrook	edith.masse@usherbrooke.ca
Scott Mckillop		scott.mckillop@lhsc.on.ca
Elka Miller	University of Ottawa	EMiller@cheo.on.ca
Adel Mohamed	Mount Sinai Hospital	adel.amohamed@sinaihealthsystem.ca

Khorshid Mohammad	University of Calgary	Khorshid.mohammad@ahs.ca
Prashanth Murthy	University of Calgary	Prashanth.murthy@ahs.ca
Poornima Murthy	Regina General Hospital	Poornima.Murthy@saskhealthauthority.ca
Alana Newman	Saint John Regional Hospital	newmanam@dal.ca
Yves G Patenaude	CHUS-Hôpital Fleurimont	Yves_gp@hotmail.com
Bruno Piedboeuf	University of Laval	bruno.piedboeuf@fmed.ulaval.ca
Nicola Proctor	Victoria General Hospital	nicola.proctor@viha.ca
Stephanie Redpath	University of Ottawa	sredpath@cheo.on.ca
James Scott	University of Calgary	James.Scott@ahs.ca
Prakesh Shah	Mount Sinai Hospital	Prakeshkumar.Shah@sinaihealth.ca
Vibhuti Shah	Mount Sinai Hospital	vibhuti.shah@sinaihealthsystem.ca
Amneet Sidhu	McMaster Children's Hospital	sidhua26@mcmaster.ca
Nina Stein	McMaster Children's Hospital	steinnina@hhsc.ca
Sumesh Thomas	University of Calgary	Sumesh.thomas@ahs.ca
Diane Wilson	The Hospital for Sick Children	diane.wilson@sickkids.ca
Nagwa Wilson	University of Ottawa	nawilson@cheo.on.ca
Justin Ryan Yuen	Janeway Children's Health Centre	Ryan.Yuen@easternhealth.ca
Hussein Zein	University of Calgary	Hussein.Zein@ahs.ca
